# Dynamic miRNA-mRNA interactions coordinate gene expression in adult *Anopheles gambiae*

**DOI:** 10.1371/journal.pgen.1008765

**Published:** 2020-04-27

**Authors:** Xiaonan Fu, Pengcheng Liu, George Dimopoulos, Jinsong Zhu

**Affiliations:** 1 The Interdisciplinary Ph.D. Program in Genetics, Bioinformatics, and Computational Biology, Virginia Tech, Blacksburg, Virginia, United States of America; 2 Department of Biochemistry, Virginia Tech, Blacksburg, Virginia, United States of America; 3 W. Harry Feinstone Department of Molecular Microbiology and Immunology, Bloomberg School of Public Health, Johns Hopkins University, Baltimore, Maryland, United States of America; University of Kentucky, UNITED STATES

## Abstract

microRNAs (miRNAs) are increasingly recognized as important regulators of many biological processes in mosquitoes, vectors of numerous devastating infectious diseases. Identification of *bona fide* targets remains the bottleneck for functional studies of miRNAs. In this study, we used CLEAR-CLIP assays to systematically analyze miRNA-mRNA interactions in adult female *Anopheles gambiae* mosquitoes. Thousands of miRNA-target pairs were captured after direct ligation of the miRNA and its cognate target transcript in endogenous Argonaute–miRNA–mRNA complexes. Using two interactions detected in this manner, miR-309-*SIX4* and let-7-*kr-h1*, we demonstrated the reliability of this experimental approach in identifying *in vivo* gene regulation by miRNAs. The miRNA-mRNA interaction dataset provided an invaluable opportunity to decipher targeting rules of mosquito miRNAs. Enriched motifs in the diverse targets of each miRNA indicated that the majority of mosquito miRNAs rely on seed-based canonical target recognition, while noncanonical miRNA binding sites are widespread and often contain motifs complementary to the central or 3’ ends of miRNAs. The time-lapse study of miRNA-target interactomes in adult female mosquitoes revealed dynamic miRNA regulation of gene expression in response to varying nutritional sources and physiological demands. Interestingly, some miRNAs exhibited flexibility to use distinct sequences at different stages for target recognition. Furthermore, many miRNA-mRNA interactions displayed stage-specific patterns, especially for those genes involved in metabolism, suggesting that miRNAs play critical roles in precise control of gene expression to cope with enormous physiological demands associated with egg production. The global mapping of miRNA-target interactions contributes to our understanding of miRNA targeting specificity in non-model organisms. It also provides a roadmap for additional studies focused on regulatory functions of miRNAs in *Anopheles gambiae*.

## Author summary

Metazoan miRNAs typically bind to partially complementary sites in their target mRNAs. The interactions between miRNAs and target RNAs are generally stage-specific and context-dependent. Thus, identification of authentic miRNA targets remains a big challenge. Target identification is even more difficult in mosquitoes where miRNA-mRNA pairing rules are poorly characterized. Using an experimental approach, this study captures thousands of endogenous miRNA-target interactions in female mosquitoes at several critical stages during adult reproduction. Analyses of the target sequences reveal how individual miRNAs accomplish their target recognition in mosquitoes. Interestingly, many mosquito miRNAs exhibit flexibility to use distinct sequences at different stages to pair with their targets, greatly altering target selectivity and expanding target repertoire of miRNAs. Drastic changes in mRNA abundance have been previously reported when adult female mosquitoes attend to varying nutritional sources and physiological demands. The temporal patterns of miRNA-target interactions obtained in this study provide new insights into the roles of miRNAs in tightly controlled gene expression associated with blood-feeding and mosquito oogenesis.

## Introduction

Mosquito-borne infectious diseases, such as malaria, dengue, chikungunya, and Zika, pose an increasing threat to public health in many tropical and subtropical regions of the world [1]. Female mosquitoes transmit the causative pathogens while taking blood from humans to acquire nutrition for egg production. Decoding complex gene regulation that governs mosquito reproduction could potentially open new avenues to develop effective disease control strategies.

Hematophagous mosquitoes have evolved regulatory mechanisms to adapt to different nutritional sources and to coordinate metabolic and reproductive processes [2, 3]. The first gonotrophic cycle in anautogenous mosquitoes consists of the previtellogenic period and vitellogenic period, separated by blood-feeding. In the early hours after eclosion, adult mosquitoes rely on nutrient reserves (mainly glycogen and lipids) accumulated during the larval stage until they could find plant nectar or fruit juice [4]. During the remaining previtellogenic period, mosquitoes use carbohydrates of nectar to synthesize trehalose for immediate usage and store surplus nutrients in the form of glycogen and lipids [5]. By 72 hours post-eclosion (PE), female mosquitoes have completed previtellogenic maturation under the control of juvenile hormone (JH) and become competent for massive yolk protein synthesis and deposition [6, 7]. The development of oocytes then enters a state-of-arrest that persists until a protein-rich blood meal is taken. Teneral reserves and energy stored during the previtellogenic period are critically important for the fate of developing oocytes [8, 9]. The vitellogenic period is controlled by 20-hydroxyecdysone (20E), the levels of which are elevated shortly after blood ingestion. A female mosquito can take in a blood meal that is greater than its body weight. Amino acids derived from blood proteins are used for the synthesis of yolk protein precursors and provide fuel to meet massive energy demand during vitellogenesis [10].

Metabolic activities in female mosquitoes thus must synchronize accordingly with interchanging energy sources. Indeed, transcriptomic analyses in the yellow fever mosquito *Aedes aegypti* have confirmed that substantial physiological changes during vitellogenesis are accompanied by significant changes in the expression of metabolic genes [6, 11]. Expression of key enzymes in glycolysis and glycogen/sugar metabolism is relatively strong in the first 24 h after eclosion and then declines considerably in the rest of the previtellogenic period [12]. Their expression is greatly up-regulated after a blood meal, exceeding the maximal mRNA levels in the previtellogenic mosquitoes. Enzymes involved in lipid metabolism exhibit a similar expression pattern in the first gonotrophic cycle [13]. In addition to transcriptional regulation, recent studies have shown that several mosquito miRNAs play important roles in blood digestion and egg production [14–19]. However, the stage-specific regulations by miRNAs and global miRNA-mRNA interaction networks have not been well explored in female mosquitoes.

miRNAs belong to a class of small non-coding endogenous RNAs that act as post-transcriptional regulators of gene expression. Mature miRNAs are loaded onto the Argonaute (Ago) protein to form the miRNA-induced silencing complex (RISC). miRNA directs RISC to its target mRNA through Watson-Crick base pairing, promoting RNA degradation and translational inhibition [20]. The nature of miRNA targeting in animals is particularly complex as canonical miRNA:mRNA pairing only requires a 6-nt seed binding (nucleotides 2–7 of miRNA), although pairing to the 3’ end of miRNA can sometimes supplement canonical sites [21]. Because of the context-dependent nature of miRNA regulation, the mere presence of a miRNA-binding site does not warrant target regulation. The ability of RISC to bind and regulate specific targets is influenced by the number and position of target sites within an mRNA [22, 23], by RNA-binding proteins that limit miRNA access to target mRNA [24, 25], and by Ago-interacting proteins or post-translational modification of Ago [26–29].

To identify miRNA-target interactions in endogenous context, biochemical approaches are increasingly used to isolate miRNA effector complexes containing miRNA-mRNA duplexes [21, 30]. High-throughput sequencing of RNAs isolated by crosslinking and immunoprecipitation (CLIP) of Ago has been applied to identify transcriptome-wide miRNA targets in developing *Caenorhabditis elegans*, mouse brain, and the fat body of *Ae*. *aegypti* mosquitoes [31–33]. However, the CLIP data failed to explicitly expose the identity of miRNA bound to a certain site. An improved CLIP method, termed CLEAR (covalent ligation of endogenous Argonaute-bound RNAs)-CLIP, has an extra ligation step to join the miRNA and its targeted mRNA in purified RISC into one chimeric RNA molecule [34]. Analysis of the chimeric miRNA-target molecules allows for the systematic identification of miRNA-target interactions [35, 36]. In the present study, we applied CLEAR-CLIP to uncover thousands of miRNA-target interactions at several key stages of mosquito reproduction. We defined pairing rules for mosquito miRNAs and illustrated stage-specific motif usage *in vivo*. Importantly, the data analyses revealed that dynamic miRNA-mRNA interactions play important roles in metabolism, energy homeostasis, and egg maturation. This work provides a comprehensive view of dynamic miRNA targeting in *Anopheles gambiae* and presents a roadmap for additional functional studies.

## Results

### Mapping the miRNA-target interactions in *An*. *gambiae* using CLEAR-CLIP

We adopted a CLEAR-CLIP protocol [34] to identify miRNA-mRNA pairing in the *An*. *gambiae* RISC. After ultraviolet irradiation, lysates of mosquito abdomens were treated with RNase I to partially trim the RNAs crosslinked to Ago1 (S1 Fig). The Ago1-RNA complexes were purified using a specific AgAgo1 antibody and incubated with T4 RNA ligase 1 to facilitate direct ligation of miRNAs and target RNAs within the same complexes. The Ago1-miRNA-mRNA complexes (>130 kDa) were then isolated by size selection on an SDS-polyacrylamide gel. RNAs associated with Ago1 were extracted and processed for RNA sequencing to detect chimeric RNA molecules.

To test the specificity of miRNA-target ligation, we performed a control experiment where T4 RNA ligase 1 was omitted in the ligation of miRNAs to target mRNAs within RISC. This omission led to a 7.47-fold decrease in the number of total chimeric reads, compared with the standard procedure (S1 Table). In addition, we carried out a separate CLEAR-CLIP experiment by adding an equal amount of *Escherichia coli* total RNA to mosquito lysates before RNase I treatment and immunoprecipitation. In the mosquito-only samples, 2.37% of chimeric reads were mapped to the *E*. *coli* genome, presumably due to high homology between some mosquito RNAs and *E*. *coli* RNA sequences or because of bacterial RNA contamination from molecular reagents used in the experiments. By contrast, in the samples spiked with the bacterial RNA, 2.78% of chimeric reads were mapped to the *E*. *coli* genome (S2 Table). The results, therefore, suggested that the vast majority of detected miRNA-mRNA chimeras resulted from ligation of the miRNAs and mRNA targets within Ago1-miRNA-mRNA complexes.

**Fig 1:**
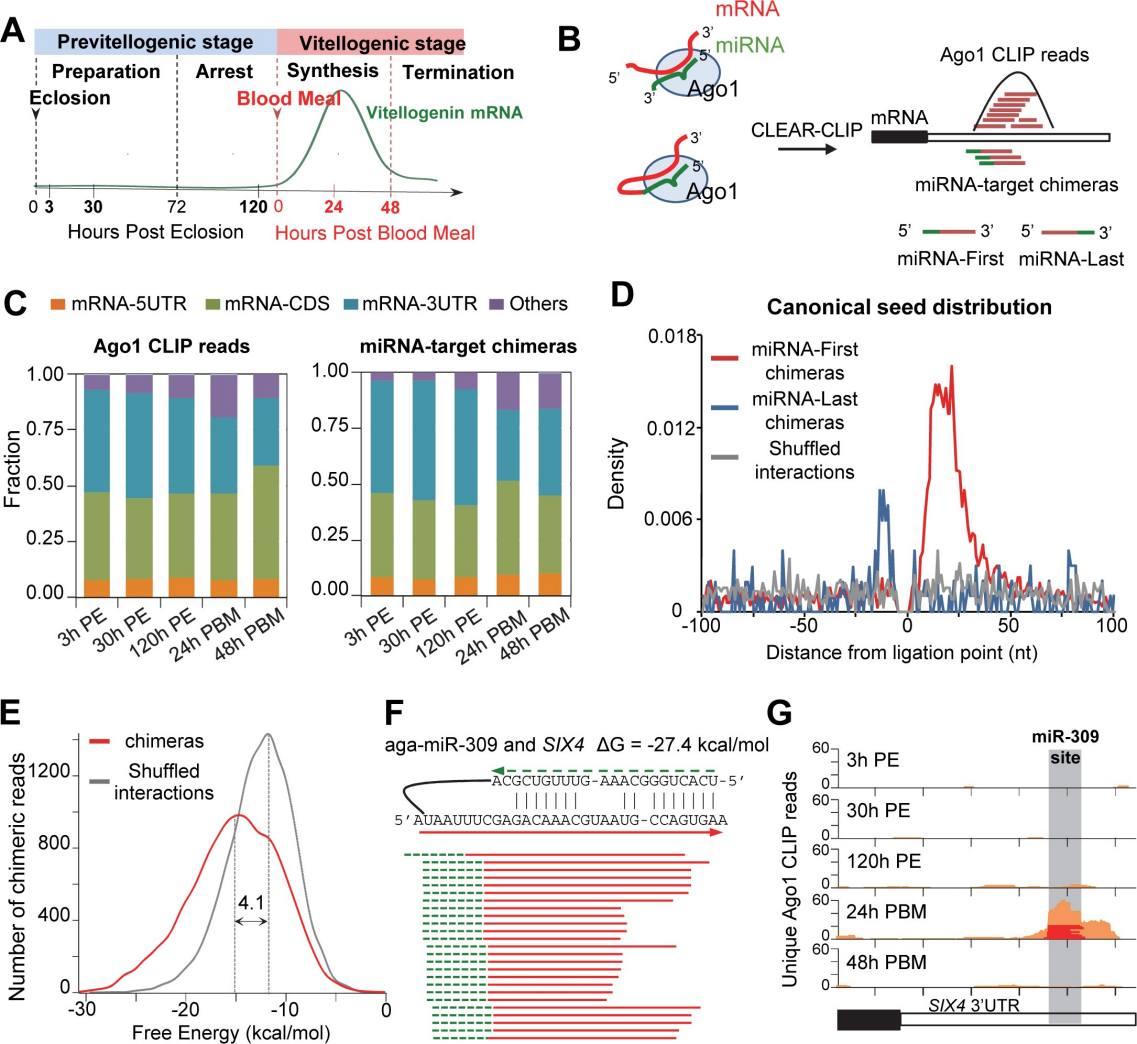
CLEAR-CLIP uncovers miRNA-mRNA interactions in *An*. *gambiae*. (A) Key events during the first gonadotrophic cycle of *An*. *gambiae*. (B) CLEAR-CLIP generated both Ago1 CLIP reads and miRNA-target chimeric reads. Clustering of the CLIP reads disclosed Ago1 binding sites. (C) Distributions of all miRNA-target chimeras and Ago1 CLIP reads in various transcript regions. Other RNAs included introns and intergenic transcripts. PE, post eclosion. PBM, post blood meal. (D) Density plot of canonical miRNA seed matches in target mRNAs relative to the ligation site in all chimeras. In the shuffled interactions (gray), each chimeric target region was randomly re-assigned to a different miRNA. (E) Predicted minimum free energy between miRNA and target mRNA found in chimeras. The shuffled interactions served as control. The difference was calculated using the median of minimum free energy predicted by RNAhybrid. (F) Examples of the miR-309-*SIX4* chimeric reads. The green dash line represents miR-309 and the red lines denote overlapping *SIX4* sequences discovered in the chimeras. The miRNA-target duplex was predicted using RNAhybrid. (G) Mapping of Ago1 CLIP reads (orange) and the miR309-*SIX4* chimeric reads (red) to the 3’ UTR of *SIX4*.

To map miRNA-mRNA interactions in *An*. *gambiae*, we collected female mosquitoes at 3 h, 30 h and 120 h post eclosion (PE), and at 24 h and 48 h post a blood meal (PBM) to perform CLEAR-CLIP experiments (Fig 1A). We obtained 305,366,254 reads from a total of 16 CLEAR-CLIP libraries (S3 Table), including miRNA-mRNA chimeras (1.70%), mRNA “single reads” (77.07%), miRNAs “single reads” (10.30%) and other noncoding RNAs (10.96%). The mRNA “single reads” harbor Ago1 binding sites and are hereinafter referred to as Ago1 CLIP reads. The chimeras were detected in two orientations: miRNA-first and miRNA-last, depending on whether miRNA was located at the 5’ end of the chimeric read (Fig 1B). When mapping to the *An*. *gambiae* transcriptome, miRNA targets derived from the chimeric reads were mostly located in the 3’-untranslated regions (3’UTR; 42.32%) and coding regions (CDS; 47.01%), but were also present in the 5’-untranslated regions (5’UTR; 10.66%). Although the distributions varied at different time points, 3’UTR and CDS remained the primary target regions of miRNAs. The Ago1 CLIP reads obtained in the same experiments exhibited similar distribution across the genic regions (Fig 1C).

Comparable to similar CLEAR-CLIP experiments using other organisms, uniquely mapped chimeras in this study were less than 3% of the total unique reads (S3 Table). Chimeras with the same miRNA and overlapping mRNA sites were clustered. In total, we have mapped 11,807 miRNA-mRNA interactions from 89,704 unique chimeric reads, involving 117 mature miRNAs and 4,755 mRNA transcripts in *An*. *gambiae* (S4 Table). Chimeric reads were combined with conventional CLIP reads from biological replicates to generate 40,919 Ago1-binding CLIP peaks. There was a significant correlation between miRNA abundance at each time point and miRNA frequency in chimeras (S2 Fig). The top five miRNAs in the miR-first chimeras, miR-8-3p, miR-14-3p, miR-989-3p, miR-34-5p, and miR-184-3p, were all known abundant miRNAs in adult female mosquitoes [37, 38].

### Validation of the miRNA-target interactions defined by CLEAR-CLIP

Several lines of evidence further indicated that the chimeras stemmed from genuine mRNA-miRNA pairing in RISC rather than from proximity-induced ligation of non-specific RNAs in solution. In 75% of miRNA-mRNA interactions that were supported by at least 9 unique tags, target sequences in the chimeric reads overlapped with an Ago1 CLIP peak (S4 Table). In addition, target sequences in the chimeras (especially miR-first chimeras) were strongly enriched for canonical seed matches to their cognate miRNAs (Fig 1D). Moreover, the formation of miRNA-mRNAs chimeras seemed to favor stable miRNA-mRNA duplexes in RISC (Fig 1E). The mean predicted free energy between miRNAs and the matched target mRNAs in chimeras was 4.1 kcal/mol lower than that in randomly matched pairs (p<0.001).

Experimentally verified miRNA-target interactions are relatively limited in mosquitoes. Our CLEAR-CLIP data retrieved a known interaction between miR-309 and homeobox gene *SIX4*, which plays an important role in ovarian development in female *Ae*. *aegypti* mosquitoes [18]. Our recent study has shown that depletion of miR-309 in female *An*. *gambiae* mosquitoes also impairs ovarian development [38]. In the current experiment, 25 unique chimeric reads comprising miR-309 and *SIX4* mRNA were detected and all came from the mosquitoes collected at 24 h PBM (Fig 1F). In the 3’ UTR of *SIX4*, an Ago1 binding site was detected predominantly at 24 h PBM; this site overlapped considerably with the miR-309 target site deduced from the chimeric reads, lending additional support to this stage-specific interaction (Fig 1G).

**Fig 2:**
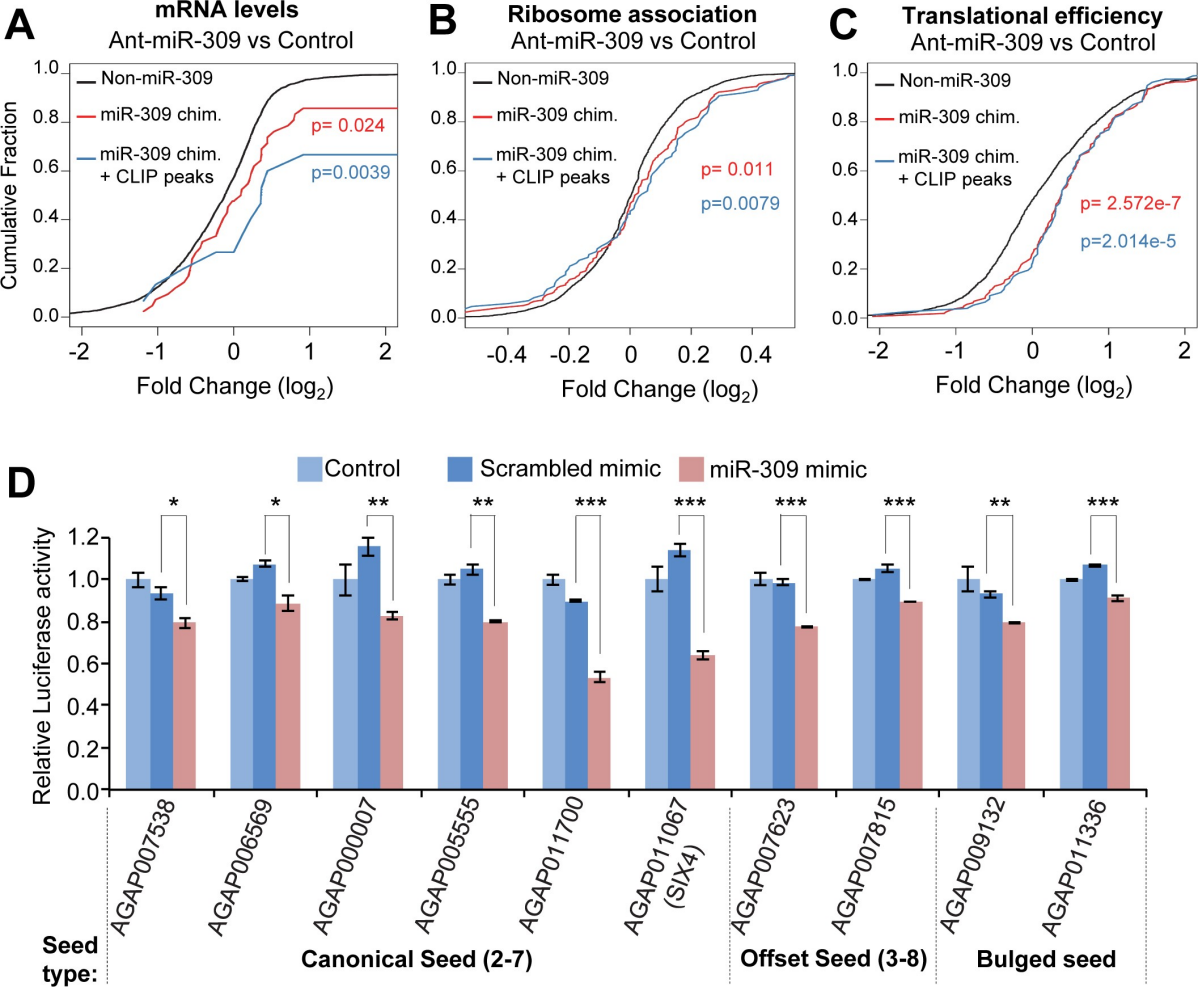
Experimental validation of the chimera-defined targets of miR-309. (A) Fold change in mRNA abundance in the ovary at 24 h PBM in *An*. *gambiae* injected with an antagomir for miR-309 verse a control antagomir. Antagomirs were injected into female adults at 2 h PE. The graph shows a cumulative distribution of the log_2_ fold change of mRNA levels for different sets of mRNAs: all targets identified in the chimeras involving miR-309 (red line), chimeras-defined miR-309 targets that were also supported by overlapping Ago1 CLIP peaks (blue line), and random transcripts which were not identified as the targets of miR-309 (black line). The difference between the distributions was analyzed by a Kolmogorov-Smirnov test. (B) Change in ribosome association of mRNA in *An*. *gambiae* after injection of antagomir-309. A scrambled antagomir sequence was used as a negative control. Ribosome occupancy was determined using ribosome footprinting. (C) Comparison of translational efficiency (TE) in *An*. *gambiae* injected with antagomir-309 or control antagomir. The TE of an mRNA was determined as ribosome occupancy normalized by its mRNA abundance. (D) Verification of the miR-309-target interactions using luciferase reporter assays. The reporter plasmids were constructed by inserting DNA fragments that contain the miR-309 target sites downstream of the *Renilla* luciferase coding region in a psiCHECK-2 vector. PCR primers used for cloning were listed in S5 Table. Results are shown as the ratio of the *Renilla* luciferase activity to the firefly luciferase activity (mean ± SD, n = 3). Statistical analyses were performed using a Student’s t-test (*, *p*<0.05; **, *p*<0.01; ***, *p*<0.001).

A total of 220 miR-309-target interactions were identified by CLEAR-CLIP assays, involving 204 mRNA transcripts. Gene Ontology (GO) and Kyoto Encyclopedia of Genes and Genomes (KEGG) analysis suggested that the miR-309 targets have important functions in post-embryonic morphogenesis and phosphate metabolic process. To validate the miR-309-involved interactions, we injected the antagomir for miR-309 (ant-miR-309) into newly emerged female mosquitoes to silence endogenous miR-309 (S3A Fig). Ovaries of female *An*. *gambiae* were collected at 24 h PBM and subjected to RNA-seq analysis. Compared with control antagomirs, ant-miR-309 significantly increased the abundance of those mRNAs that bear chimeras-defined miR-309 target sites (p = 0.024, Kolmogorov-Smirnov test) (Fig 2A). The increases were more robust (p = 0.004) for the target genes that were supported by both chimeric reads and overlapping Ago1 binding sites (Fig 2A). In line with the previous study in *Ae*. *aegypti* [18], both RNA-seq and quantitative RT-PCR confirmed that injection of ant-miR-309 caused a marked increase in the *SIX4* transcript at 24 h PBM (S3B and S3C Fig).

In parallel, we performed ribosome footprinting to evaluate translational repression conferred by the binding sites of miR-309. Compared with control antagomirs, ant-miR-309 significantly enhanced ribosome associations of the transcripts harboring miR-309 target sites (Fig 2B). Translation efficiency (TE), which is defined as ribosome footprint density normalized to underlying mRNA abundance, also increased significantly after injection of anti-miR-309 (Fig 2C). Thus, the results indicated that miR-309 modulates the expression of its targets at 24 h PBM via both translational repression and mRNA decay.

To further assess the regulatory potential of the identified interactions, ten miR-309 targets that exhibited various pairing patterns with the seed sequence of miR-309 were selected for luciferase reporter assays (S3D Fig). DNA fragments containing the miR-309 binding sites were cloned individually into a luciferase reporter vector. HEK293 cells were transfected with the reporter constructs alone or together with miRNA mimics. Remarkably, the normalized luciferase activities of all 10 reporter constructs were significantly down-regulated by the miR-309 mimic, compared with the control mimic (Fig 2D). The miR-309 target site in the 3’ UTR of *SIX4* mRNA conferred relatively strong repression of the reporter gene by the miR-309 mimic. Collectively, the miRNA perturbation experiments and reporter assays suggested that miRNA-mRNA chimeric reads, generated by CLEAR-CLIP and bolstered by overlapping Ago 1 binding sites, can reliably identify authentic miRNA-mRNA interactions *in vivo*.

### Regulation of *kr-h1* by let-7 during egg development

**Fig 3:**
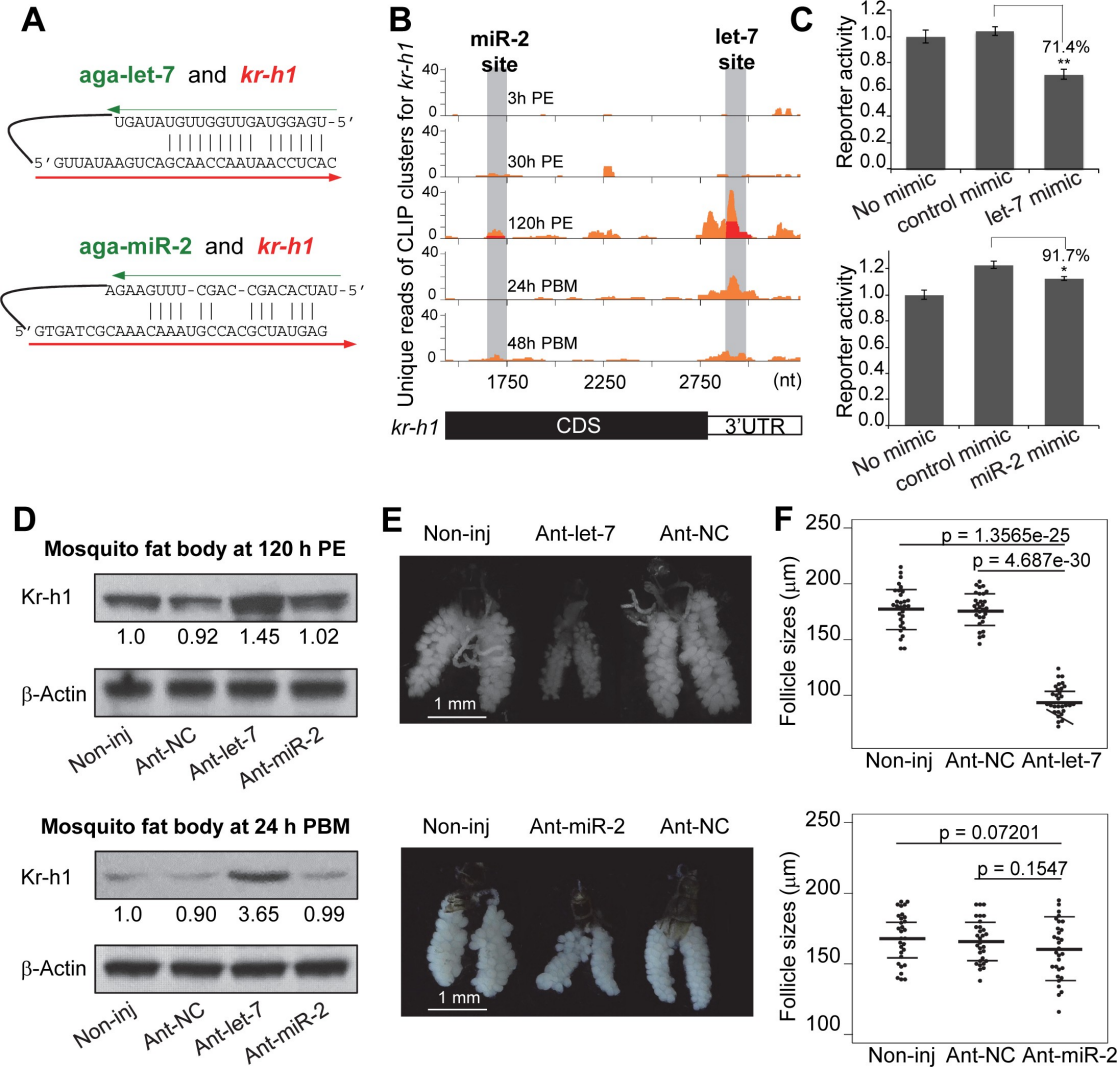
Modulation of *kr-h1* expression by let-7 is essential for normal egg development. (A) The interactions of *kr-h1* with let-7 and miR-2 defined by chimeric reads. (B) miRNA target sites in *kr-h1*. Distribution of Ago1 CLIP peaks (orange) was plotted along the *kr-h1* transcript. Overlapped miRNA-target chimeras were labeled in red. (C) Luciferase reporter assays to test the interaction of miR-2, let-7 with *kr-h1*. DNA fragments bearing putative binding sites were cloned into psiCHECK-2. Three biological replicates were used for measurements. Statistical analyses were performed using a Student’s t-test (*, *p*<0.05; **, *p*<0.01). (D) Expression of Kr-h1 after antagomir injection. Specific (Ant-let-7, Ant-miR-2) and control (Ant-NC) antagomirs were injected into adult female mosquitoes at 12 h after adult emergence. Western blot was performed to measure the Kr-h1 protein at 120 h PE (before taking a blood meal) and at 24 h PBM after antagomir treatment. Non-inj, the uninjected control. (E) Oocyte development in the antagomir-treated mosquitoes at 24 h PBM. (F) Follicle sizes of the antagomir-treated mosquitoes at 24 h PBM. Thirty individuals in each group were measured using the Leica Application Suite (v4.5). Statistical analyses were performed using a Student’s t-test.

Another interaction that was experimentally confirmed in this study was the regulation of *krüppel-homolog 1* (*kr-h1*) by let-7. Kr-h1, a juvenile hormone-induced transcription factor, has been shown to play a pivotal role in egg maturation in *Ae*. *aegypti* [39]. Our CLEAR-CLIP data suggested that let-7 and miR-2 directly regulate the expression of *kr-h1* in adult female *An*. *gambiae*. Both let-7-*kr-h1* and miR-2-*kr-h1* chimeras were detected from mosquitoes collected at 120 h PE; let-7 seemed to target *kr-h1* more vigorously than miR-2, based on the number of chimeric reads (Fig 3A and 3B). The target sites of let-7 and miR-2 were located in the 3’UTR and CDS of *kr-h1*, respectively.

Interactions of *kr-h1* with miR-2 and let-7 were verified in luciferase reporter assays. Compared with scrambled control miRNAs, miR-2 and let-7 demonstrated significant repression of the luciferase reporter genes that carried their cognate target sequences identified in *kr-h1* (Fig 3C and S4 Fig). To evaluate the contribution of both miRNAs to *kr-h1* expression *in vivo*, we measured the Kr-h1 proteins after knocking down endogenous let-7 or miR-2 by injecting specific antagomirs into newly emerged mosquitoes. At 120 h PE, the levels of Kr-h1 increased by 45% after injection of ant-let-7 while the protein levels remained essentially unchanged after the ant-miR-2 treatment (Fig 3D). At 24 h PBM, ant-miR-2 showed no significant impact on the protein levels of Kr-h1 while the ant-let-7 treatment increased the expression of Kr-h1 by 3.65 fold, compared to the control (Fig 3D). Although the results suggested that let-7 is the major regulator of *kr-h1*, it is possible that the decrease in miR-2 levels, after injection of ant-miR-2, did not reach a critical threshold to cause any significant change in the expression of *kr-h1* (S5 Fig). In the let-7-depleted female mosquitoes, primary follicles failed to increase in size after a blood meal, indicating that the development of oocytes was stalled (Fig 3E and 3F). In contrast, knockdown of the miR-2 expression has no significant effect on oocyte development after blood ingestion (Fig 3E and 3F). Different roles of let-7 and miR-2 in the regulation were also validated by the injection of miRNA mimics. The let-7 mimic, not the miR-2 mimic, caused a considerable decrease in Kr-h1 at 120 h PE and 24 h PBM and suppressed oocyte development after blood feeding (S6 Fig). Taken together, these results demonstrated that precise regulation of *kr-h1* by let-7 is essential for egg maturation in *An*. *gambiae*, reminiscent of a similar role of this interaction in adult reproduction in migratory locust *Locusta migratoria* [40].

### Diverse and stage-specific miRNA target site recognitions in *An*. *gambiae*

**Fig 4:**
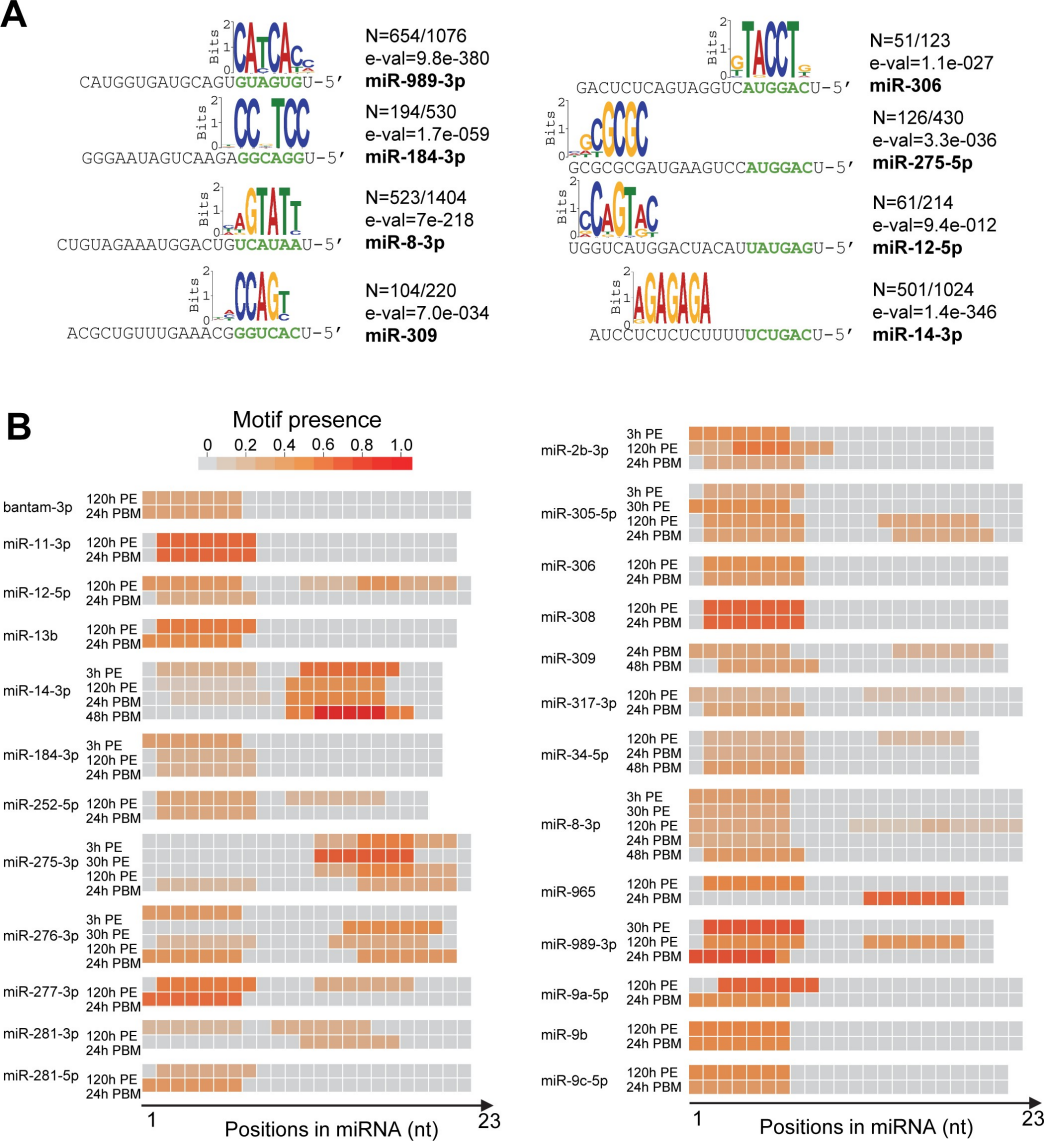
Sequence motifs enriched in miRNA targets. (A) Significantly enriched motifs for individual miRNAs were derived from chimeras-defined target sequences using the MEME tool. The MEME analysis was performed with the default 0-order Markov model to define 7-mer motifs. Shown here are motifs identified for representative miRNAs. N, number of motifs found/total number of targets analyzed; e-val, e-value of the motif reported by MEME. Seed sequences of miRNAs are in green. (B) *De novo* analysis of cognate miRNA-complementary-enriched 7-mer motifs in the chimeras that were detected at different time points. miRNAs with at least 30 unique chimeras-defined targets at a single time point were selected for the motif usage analysis using Homer. The motifs were plotted as a heatmap across the miRNA, with color intensity indicating the proportion of motifs in target sequences.

The experimentally identified miRNA-target interactions open the door to deciphering the rules of miRNA targeting in mosquitoes. Our CLEAR-CLIP analysis indicated that abundant miRNAs generally formed chimeras with multiple target mRNAs. To characterize the base-pairing patterns of miRNA-target interaction, we searched for overrepresented sequence elements in all the targets discovered for each miRNA. For most of the top expressed miRNAs, highly enriched sequence motifs emerged as complementary to the extended miRNA seed region (nucleotides 1–8 of miRNA) (Fig 4A and S7 Fig). Close inspection of the enriched motifs indicated that imperfect seed matches are quite common in mosquitoes. miR-184-3p displayed substantial mismatches at nucleotide 5 in the 5' region in pairing with its targets; for miR-309, considerable mismatches occurred at nucleotide 8 (Fig 4A). In contrast, many miRNAs seemed to favor seed-independent non-canonical pairing to determine their target specificity. Motifs found in the targets of miR-14-3p and miR-275-5p suggested that those two miRNAs prefer to use their 3’ regions for target recognition (Fig 4A). Interestingly, miR-275 and miR-306, which belong to the same family and share identical seed sequence, used opposite ends of miRNA to pair with their cognate targets (Fig 4A).

Next, unbiased *de novo* motif (7-mer) analyses were performed with miRNAs that participated in over 50 unique interactions to compare the enriched motifs for individual miRNAs at different time points. Some miRNAs, such as bantam-3p, miR-11-3p, miR-306, miR-308, miR-9b, and miR-9c-5p, consistently used the same sequences to recognize their targets at several time points (Fig 4B). Many miRNAs exhibited 1- or 2-nt shifts in their recognition sequences from one time point to another. For instance, miR-184-3p primarily used nucleotides 1–7 at 3 h PE for target recognition (Fig 4B). However, this miRNA relied on nucleotides 2–8 instead at 120 h PE and 24 h PBM. Moreover, miR-9a-5p appeared to use nucleotides 3–9 for pairing with its targets at 120 h PE but switched to nucleotides 1–7 at 24 h PBM (Fig 4B). This motif shift of miR-9a-5p was accompanied with drastic changes in its targets from 120 h PE to 24 h PBM (S8 and S9 Figs). Among 65 targets of miR-9a-5p detected at 24 h PBM, only 8 were also regulated by miR-9a-5p at 120 h PE. One possible reason for the motif shifts is the existence of miRNA isoforms. However, only miR-2b showed an isoform with a 2-nt truncation at the 5’ end, explaining the 2-nt motif shifting at 120 h PE. miRNA isoforms were not detected in other cases.

Sequences in the middle or 3’ end of some miRNAs seemed to also contribute to target recognition, according to the enriched motifs in miRNA targets. At 3 h PE, 10.7% of miR-14-3p targets carried sequences complementary to its extended seed region while 50.4% of the targets harbored a motif that matched to nucleotides 12–18, with an additional 9.9% of the miR-14-3p-target interactions necessitating base pairing of both regions (S10A Fig). Later at 48 h PBM, nucleotides 11–19 accounted for 79.1% of target recognition by miR-14-3p. Other miRNAs also displayed striking stage-specific changes in using their different sequences for target recognition. miR-276-3p mainly relied on nucleotides 1–7 for pairing with its targets at 3 h PE and switched to nucleotides 15–21 at 30 h PE (S10B Fig). At 120 h PE and 24 h PBM, miR-276-3p depended on the sequences at both 5’ and 3’ ends. The diverse pairing patterns of miR-14-3p and miR-276-3p were consistent with their vastly different targets retrieved at each stage during adult reproduction (S8 and S9 Figs). Collectively, the analyses revealed extensive non-canonical interactions between mosquito miRNAs and their mRNA targets, as well as stage-specific recognition patterns of some miRNAs.

### Regulation of mosquito metabolism by miRNAs

**Fig 5:**
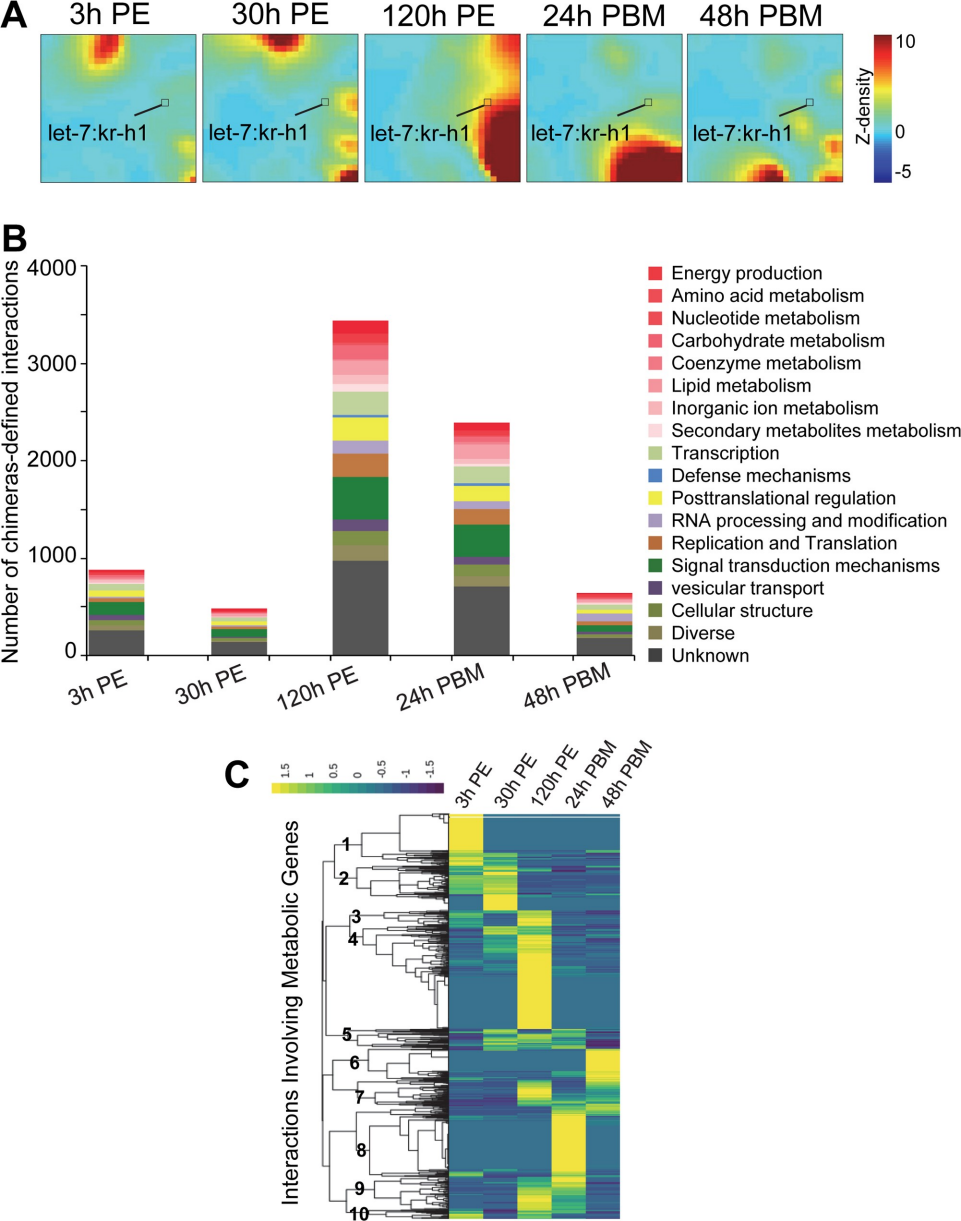
Massive and dynamic miRNA-target interactions in mosquito reproduction. (A) Patterns of miRNA-target interactions. The chimeras-supported and normalized Ago1CLIP peaks were analyzed using Gene Expression Dynamics Inspector (GEDI). The interaction data were clustered and transformed into a two-dimensional self-organizing map for individual time points. Each tile at the same position in all the maps corresponds to a group of miRNA-target interactions that share similar patterns throughout the 5 time points during adult reproduction. The color scale denotes the heights of Ago1 CLIP peaks. The small square represents the group that includes let-7-*kr-h1* interaction. (B) Functional annotation of miRNA targets at each time point. (C) Regulation of mosquito metabolic genes by miRNAs in adult reproduction. The clustering analysis displays discrete patterns of interactions between mosquito miRNAs and genes involved in metabolism. The heights of peaks were z-normalized.

We selected 7,025 chimera-defined interactions that were supported by Ago1 CLIP peaks to investigate the dynamics of miRNA-involved regulation during mosquito reproduction. To compare miRNA-mRNA interactions in different stages, the chimera-supported Ago1 binding peaks were normalized to mRNA abundance to take into consideration changes of mRNA levels. The normalized Ago1 CLIP signals were then analyzed using the self-organized map (SOM) algorithm to group large sets of interactions with similar patterns to simplify visualization. The 2-dimensional gridded heatmap revealed the stage-dependent and dynamic nature of miRNA-mRNA interactions in adult female mosquitoes. The similar patterns at 3 h PE and 30 h PE (R = 0.69) implied that many miRNAs-involved regulations persisted during the early previtellogenic stage (Fig 5A). Dramatic pattern shifts occurred later when female mosquitoes proceeded from preparatory phase (30 h PE) to the state-of-arrest (120 h PE), to active vitellogenic synthesis (24 h PBM), and to termination of vitellogenic stage (48 h PBM). Gene regulation by miRNAs was most prevalent at 120 h PE and 24 h PBM (Fig 5B).

Functional annotation of the identified miRNA target genes indicated that miRNAs in adult female mosquitoes modulate diverse molecular processes, including various metabolic functions (Fig 5B). The hierarchical clustering of these metabolism-related interactions revealed distinct interaction patterns at different stages during the first gonotrophic cycle (Fig 5C), suggesting that miRNAs make a major contribution to the precise regulation of energy homeostasis to deal with alternating energy sources and physiological demands in adult female mosquitoes.

**Fig 6:**
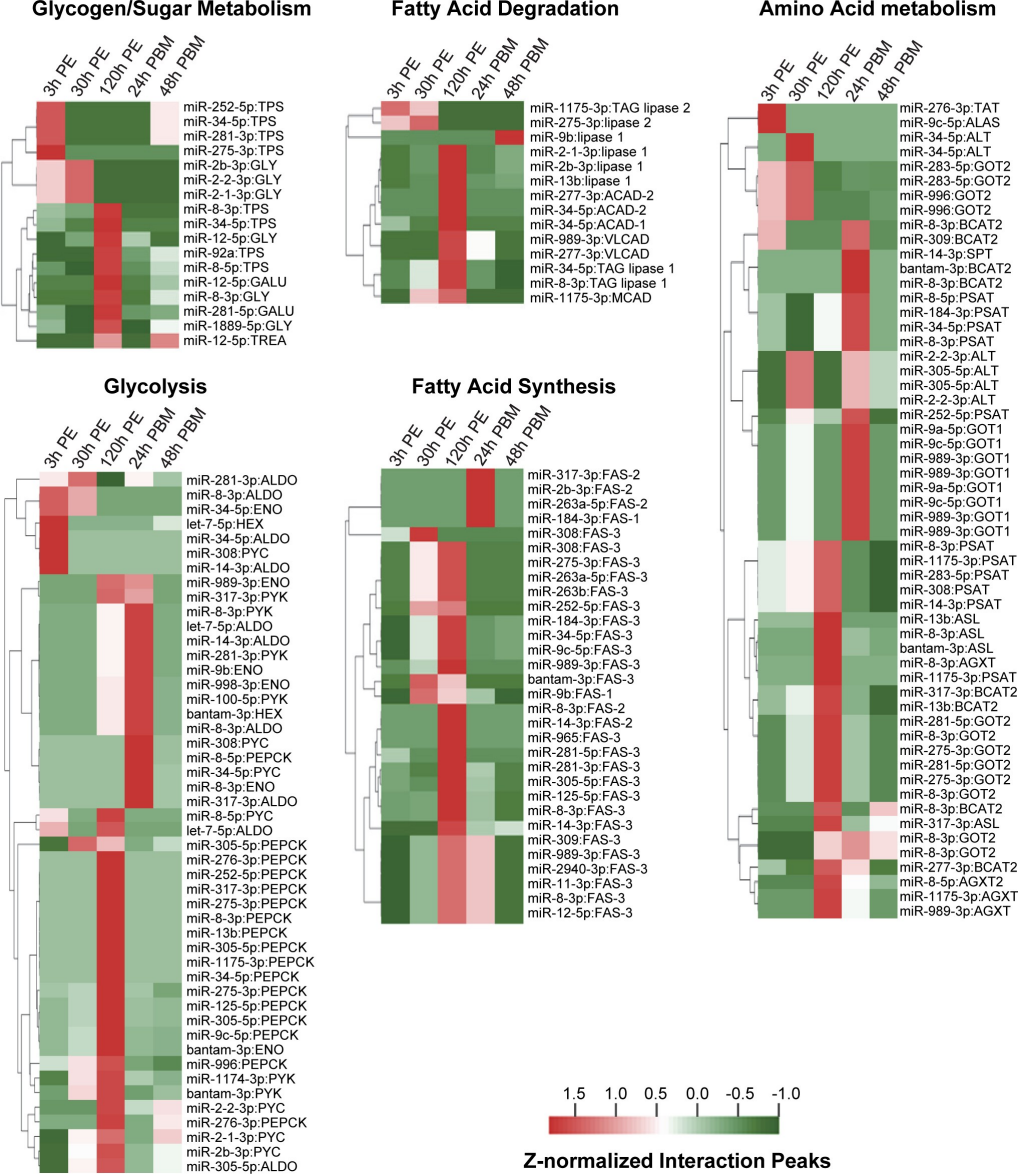
Potential roles of miRNAs in the regulation of carbohydrate, lipid and amino acid metabolism in adult mosquitoes. The chimeras-supported and normalized Ago1CLIP peaks were analyzed. The heatmaps show the patterns of miRNA interactions involving various metabolic genes. Dendrograms generated by hierarchical clustering of target genes from representative pathways are also provided. The full names of selected target genes are described in S6 Table.

The genes encoding glycogen/sugar metabolism (4 out of 17 enzyme-coding genes) and glycolysis (6 out of 28 enzyme-coding genes) exhibited particularly pronounced fluctuation in their interactions with miRNAs (Fig 6). Hours after eclosion, adult female mosquitoes reared in the laboratory generally rely on provided sucrose solution to meet their energy demand. Ingested sucrose is digested by α-glucosidases and converted into trehalose, the principal hemolymph sugar that provides circulating energy in flight and host-seeking. Extra sugars are converted into somatic energy reserves in the form of lipids and glycogen to support the imminent egg maturation following a blood meal. The miRNA regulation of glycogen metabolism and glycolysis, identified in this study, seems to be consistent with the stage-specific metabolic alteration associated with sugar feeding. Expression of trehalose-6-phosphate synthase (TPS) was inhibited by miR-34/-252/-275/-281 at 3 h PE, presumably before sugar feeding (Fig 6). Glycogen phosphorylase (GLY) catalyzes the rate-limiting step in glycogenolysis. Its expression was repressed first at 30 h PE by miR-2b-3p and miR-2-3p, and later at 120 h PE by miR-8-3p/-12-5p/-1889-5p (Fig 6). UTP-glucose-1-phosphate Uridylyltransferase (GALU), which catalyzes the formation of UDP-glucose for glycogen synthesis, was under the control of miR-12-5p and miR-281-5p at 120 h PE. The suppression of GALU was in accordance with the observed slowdown of glycogen synthesis once glycogen reaches a certain level in sugar-fed female mosquitoes [12]. Several key enzymes in glycolysis, such as enolase (ENO), pyruvate kinase (PYK), and pyruvate carboxylase (PYC), were primarily targeted by various miRNAs at 120 h PE and 24 h PBM (Fig 6). Notably, the phosphoenolpyruvate carboxykinase (PEPCK) gene, which governs a rate-controlling step of gluconeogenesis, was vigorously targeted by numerous miRNAs at 120 h PE (Fig 6).

In the sugar-fed mosquitoes, TAG is built up and stored as an energy reserve in intracellular droplets. After blood-feeding, the lipid droplets are diminished and TAG is mobilized to support oocyte development. Our study indicated that miRNA regulation of the genes involved in lipid metabolism displayed stage-specific manners. Enzymes required for fatty acid degradation, such as TAG lipase 1, acyl-CoA dehydrogenases (ACAD), very long-chain acyl-CoA dehydrogenase (VLCAD), were strongly regulated by miRNAs at 120 h PE to repress their gene expression (Fig 6). These interactions disappeared after blood ingestion. On the other hand, the three fatty acid synthase genes (FAS-1, FAS-2, and FAS-3) were the targets of multiple miRNAs. Inhibition of FAS-3 took place primarily at 120 h PE while strong repression of FAS-2 occurred at both 120 h PE and 24 h PBM. These data implied that miRNAs play a regulatory role in lipid metabolism during mosquito reproduction.

Drastic metabolic changes occur upon adult female mosquitoes take a blood meal. Generally, only 10–15% of the blood-derived amino acids are used by female mosquitoes to synthesize egg proteins [41]. The rest of amino acids is mostly deaminated and the carbon skeletons are used for instant energy production, synthesis of egg lipid, and building energy reserves. Expression of alanine-glyoxylate aminotransferase (AGXT), an aspartate aminotransferase (GOT2), and argininosuccinate lyase (ASL, a key enzyme in the urea cycle) was specifically repressed by various miRNAs at 120 h PE. The gene encoding another aspartate aminotransferase (GOT1) was selectively repressed by miRNAs at 24 h PBM, while the transcripts of phosphoserine aminotransferase (PSAT) and a branched-chain amino acid aminotransferase (BCAT2) were targeted by several miRNAs at both 120 h PE and 24 h PBM. The results implied that miRNAs participate in repressing the expression of amino acid metabolic enzymes in sugar-fed mosquitoes and that the de-repression of metabolic genes for individual amino acids may occur at different paces. Taken together, this study suggests that developmental patterns of miRNA-mRNA interactions define important regulatory roles of miRNAs in metabolism and energy homeostasis during mosquito reproduction.

## Discussion

In this study, we performed CLEAR-CLIP experiments to generate a miRNA-mRNA interaction network during egg maturation in adult female mosquitoes. In addition to revealing target sites of miRNAs, this approach provided temporal patterns of individual miRNA-target interactions. Using miR-309 as an example, we experimentally validated its interaction with the targets identified by CLEAR-CLIP, including the miR-309-*SIX4* interaction which has been well characterized in *Ae*. *aegypti*. The regulation of *SIX4* by miR-309 in *An*. *gambiae* took place primarily at 24 h PBM, coinciding with a similar pattern in *Ae*. *aegypti* [18]. Moreover, we used the let-7-*kr-h1* interaction to demonstrate that temporal patterns of interactions are instrumental for the functional elucidation of miRNAs. Furthermore, we used the CLEAR-CLIP data to compare the miRNA-target interactions in *An*. *gambiae* and *Ae*. *aegypti*. Ago1 binding sites have been identified by CLIP in the fat body of *Ae*. *aegypti* before and after blood feeding [33]. For those *Ae*. *aegypti* genes with identified Ago1 CLIP peaks, about 70% of their orthologs in *An*. *gambiae* also harbored Ago1 binding sites at the corresponding time points (S11 Fig). Given that we used mosquito abdomens consisted of several different tissues in the current study, the miRNA-mRNAs interactions appear to be highly conserved between the two mosquito species.

It came to our attention that the CLEAR-CLIP experiments failed to retrieve some previously identified miRNA-target interactions in *An*. *gambiae*, e.g. the regulation of *secreted wingless-interacting molecule* (*swim*) by miR-8 and the interaction between miR-1174 and the serine hydroxymethyltransferase gene [16, 17]. This implied that although the CLEAR-CLIP experiment generated substantial miRNA-mRNA chimeras, the miRNA-target interaction map that it produced was far from complete. Therefore, while the chimeric reads provided unambiguous miRNA-target interactions, the Ago1 CLIP peaks were a more reliable measurement of miRNA-target interactions at different stages during the first gonotrophic cycle. The limitation of this experiment mostly stemmed from low efficiency in RNA ligation under current experimental conditions (S3 Table). The low efficiency in RNA ligation is in accord with other published studies using the CLEAR-CLIP approach. In the studies of mouse brain or *Caenorhabditis elegans*, miRNA-mRNA chimeras also accounted for 1–3% of total reads [34–36]. Chimeras recovered in the current study preferentially contained shorter mRNA sequences (~42 nt), suggesting that proper trimming of mRNA cross-linked to Ago1 is crucial for chimera formation. Further optimization of the protocol is needed to improve coverage of global miRNA regulation of gene expression.

Mapping of miRNA-target interactions creates an opportunity for deciphering the pairing rules of mosquito miRNAs. The vast majority of miRNAs preferentially bind their targets through imperfect base-pairing with their seed region. Other miRNAs, however, rely on their central or 3’ region for target recognition. While paired seed sites are occasionally supplemented by the seed-distal 3’ pairing, the targets in many chimeric reads often show significant match exclusively with the seed-distal region, suggesting that sometimes seedless pairing is a major determinant of Ago target specificity. Some miRNAs, such as miR-14-3p, utilize their seed region, distal region or both to recognize different targets at the same reproductive stage. Although we cannot rule out the possibility that distinct recognition patterns occur in a tissue-specific manner, the flexible targeting mechanism greatly expands the target repertoire of a miRNA. Moreover, while miR-306 exploits canonical miRNA targeting, miR-275-5p (a member of the same miRNA family) shares the same seed sequence but is reliant on its 3’ end for target recognition. Further study is needed to investigate the role of miR-275-5p seed in its seedless pairing and to elucidate how miRISC recognizes miRNA targets that lack seed complementarity.

Another important observation of this study is the varying target recognition patterns of the same miRNAs at different stages in adult mosquitoes. The alterations include small shifting of recognition sites (1–2 nt), substitution by a distal region of miRNA, and addition of supplementary pairing. Sequences of miRNAs collected at different stages were largely identical, suggesting that the observed pattern changes may not be accredited to alternative miRNA processing. Nevertheless, the changes in pairing patterns of miRNAs led to drastic switches of miRNA targets at different stages in the first gonotrophic cycle, contributing to the detected dynamic miRNA-mRNA interactions. This was confirmed by target analyses of miR-9a-5p, miR-14-3p, and miR-276-3p (S8 Fig), and may also be extrapolated to other mosquito miRNAs. This strategy seemed to allow individual mosquito miRNAs to regulate a diverse array of target genes, in a stage-specific manner, especially when nutritional sources and physiological demands undergo significant changes in adult reproduction.

There is a growing body of evidence that target binding and silencing efficacy is governed by several local and global determinants [42]. Some miRNAs may possess an intrinsic ability to use various regions for the recognition of different targets. Alterations in overall target abundance and target repertoire at different stages may force these miRNAs to change their recognition patterns. Additionally, many target mRNAs carry multiple miRNA sites that overlap or are in close vicinity. Binding of a miRNA to a target site may be either enhanced or repressed by the changing miRNA milieu [43, 44]. Moreover, miRNA targeting activity has been recently reported to be influenced by the phosphorylation of Ago proteins in response to other cellular signals [45]. miRNA-target interactions are also modulated by other RNA binding proteins, the binding of which to the target mRNA exposes or occludes specific miRNA target sites [46, 47]. Drastic physiological changes take place during adult reproduction in female mosquitoes. Alterations of the abovementioned determinants in specific biological contexts could contribute to the observed changes in target recognition patterns of mosquito miRNAs. More studies are needed to elucidate mechanistic details of the pattern change.

miRNA-mRNA interactions that are defined by chimeras are particularly valuable for comprehensive analysis and functional interpretation of miRNAs. In this study, about 100 interactions were identified on average for each miRNA; the miRNA targets illustrated diverse roles of individual miRNAs in various biological processes. miR-8-3p, for example, is ubiquitously abundant in mosquitoes [17, 38, 48] and *D*. *melanogaster* [49, 50]. The CLEAR-CLIP experiment identified ~1,700 mRNA interactions with miR-8-3p in adult female mosquitoes. The target genes of miR-8-3p were involved in a variety of functions including lipid localization, regulation of cellular component size, wing disc development and glycolysis/gluconeogenesis (S12 Fig). This is in agreement with the reported roles of miR-8-3p in regulating lipid accumulation in *Ae*. *aegypti* [17] and affecting body size and metabolism in *D*. *melanogaster* [49]. Injection of miR-8-3p mimic into newly emerged female *An*. *gambiae* indeed substantially reduced the accumulation of TAG in sugar-fed adults before blood-feeding (S13 Fig). The observed phenotype is likely an accumulative effect of the misexpression of multiple miR-8-3p target genes.

The dynamic miRNA-mRNA interactomes implied that miRNAs regulate many stage-specific functions in adult mosquitoes. Mosquitoes are highly dependent on available nutrients to support biochemical processes required for reproduction. Nutrient mobilization and energy homeostasis are constantly adjusted to their nutritional and metabolic status to meet the fluctuating energy demand of a reproducing female mosquito. Our results suggested that miRNAs contribute greatly to this metabolic reprogramming. During the previtellogenic stage, female mosquitoes use plant sugars to build their energy storage in the forms of glycogen and TAG for imminent egg production. After a blood meal, in addition to the blood-derived nutrients, glycogen and TAG stored in the fat body are mobilized and depleted to meet tremendous energy expenditure required for oocyte maturation. Accordingly, both carbohydrate and lipid metabolism exhibit periodic changes throughout the mosquito reproductive cycle to adapt to varying nutrient sources and energy requirements. Many genes encoding key enzymes in glycolysis and lipid catabolism are transcriptionally downregulated at the end of the previtellogenic stage but show a significantly enhanced expression after blood ingestion (S14 Fig). These gene expression patterns in female mosquitoes are under the control of juvenile hormone and 20-hydroxyecdysone [12, 13, 51, 52]. Our study revealed that these genes were further repressed by miRNAs to diminish glycolysis and lipid degradation at 120 h PE and that these miRNA regulations vanished shortly after blood feeding. Interestingly, some enzymes required for glycogen and lipid synthesis were regulated by miRNAs in a similar fashion. Thus, fine-tuning of enzyme expression by miRNAs may confer precision to the temporal coordination of metabolism and maintain energy reserves at the desired level before a blood meal.

In summary, this study shows that miRNA-mRNA interaction maps vary in different biological settings. It highlights the importance of target validation under physiological conditions to appreciate miRNA function *in vivo*. The identified miRNA-mRNA interactions not only supply a roadmap for the functional investigation of mosquito miRNAs, but also provide valuable datasets to train *in silico* algorithms to significantly improve their predictive power.

## Methods

### Ethics statement

This study was carried out in strict accordance with the recommendations in the Guide for the Care and Use of Laboratory Animals of the National Institutes of Health. The use of mice in this study has been approved by the Virginia Tech Institutional Animal Care and Use Committee (approval number: 17–162).

### Mosquito rearing and tissue collection

*Anopheles gambiae* (G3 strain) mosquitoes were reared and maintained as previously described [53]. Newly emerged mosquitoes were fed with a 10% sucrose solution. Adult females were blood-fed five days after eclosion using anesthetized mice. Mosquitoes were collected at 3 and 30 h PE, and at 0, 24, and 48 h PBM. Abdomens of the mosquitoes were flash-frozen in liquid nitrogen and stored at -80°C until use.

### Ago1 CLEAR-CLIP

Polyclonal antibody against *An*. *gambiae* Ago1 was produced by Fu et al. [38]. The CLEAR-CLIP libraries were prepared as previously described [34]. Briefly, abdomens from 100 female mosquitoes were ground in liquid nitrogen and UV-irradiated at a dose of 400 mJ/cm^2^ for three times. Following tissue lysis, lysates were centrifuged at 15,000 g for 15 min at 4°C. The supernatant was treated with 30 units of RQ1 DNase (Promega) and 2 units of RNase I (Ambion) for 3 min at 37°C. Ago1 antibody was immobilized on Protein A Dynabeads (Invitrogen) and incubated with the clear lysate for 60 min at 4°C. Precipitates were stringently washed twice with High-Salt buffer (50 mM Tris-HCl pH 7.5, 0.8 M NaCl, 0.5% NP-40, 2.5% glycerol, 5 mM β-mercaptoethanol), twice with Low-Salt buffer (50 mM Tris-HCl pH 7.5, 0.3 M NaCl, 0.5% NP-40, 2.5% glycerol, 5 mM β-mercaptoethanol), and twice with PNK (polynucleotide kinase) buffer (50 mM Tris-HCl pH 7.5, 10 mM MgCl_2_, 0.5% NP-40, 50 mM NaCl, 5 mM β-mercaptoethanol). RNAs on the beads were phosphorylated with T4 PNK (NEB) at 20°C for 150 min, and chimera ligation was then performed overnight at 16 °C with T4 RNA Ligase 1 (0.625 U/μl, NEB) in a 160 μl reaction. The 3'-phosphate was removed with Thermosensitive Alkaline Phosphatase (Promega) at 20°C for 45 min. Radiolabeled adenylated 3'-linker (5'-rAppAGATCGGAAGAGCACACGTCT-3') was prepared with Mth RNA-Ligase (NEB). The 3'-linker was ligated to the RNAs on the beads using truncated RNA Ligase 2 K227Q (NEB) at 16 °C for 6 h. After elution with NuPAGE LDS buffer (Life Technology), the Ago1-miRNA-mRNA complex (>130 kDa) was separated by SDS-PAGE (4–12% bis-tris) and transferred to a nitrocellulose membrane. The Ago1-bound RNAs were extracted from the membrane slice by proteinase K digestion (Ambion) and ligated with the 5'-RNA linker, followed by RT-PCR. The PCR amplification was performed as previously described [38], with an optimized condition to incorporate sequencing index (NEB) into each Illumina library.

### *De novo* transcriptome assembly

Mosquito abdomens were collected at the same time points as in the CLEAR-CLIP experiments. mRNAs were extracted and subjected to RNA-seq analysis. Transcriptome shotgun assembly was completed following the PASA pipeline with default parameters [54]. *An*. *gambiae* transcript sequences used for mapping of miRNA targets have been deposited at DDBJ/ENA/GenBank under the accession GIBN00000000.

### Identification of miRNA-mRNA chimeras

Paired-end sequencing of the CLEAR-CLIP libraries was conducted at Virginia Biocomplexity Institute using the Illumina HiSeq platform with a read length of 100 bp. All RNA sequencing data have been deposited in the NCBI SRA database under accession number SRP147279. Sequencing reads were processed to remove low-quality reads and trim 3’ adapter sequences using Flexbar [55]. A degenerate 3-nt barcode was included in the 5'-RNA linker to reduce preferential PCR duplication. These random barcodes were trimmed before mapping to *An*. *gambiae* transcripts. Reads shorter than 16 nt were discarded and reads with identical sequences were collapsed for further analysis. We searched miRNA sequences in the cleaned reads by using BLAST (v2.3.0) with e-value of less than 0.4 [56]. The remaining sequences next to miRNAs in the reads were extracted for mapping to the annotated *An*. *gambiae* transcripts with BLAST. Reads mapped to rRNA, tRNA and miRNA genes were removed.

### Peak calling of CLIP clusters

Peak calling of CLIP clusters was performed as previously described by Zisoulis et al. [32], using pooled reads from biological replicates of each time point. Overlapping reads were grouped as a single cluster. Cubic spline interpolation (Scipy, http://www.scipy.org/) was then carried out to determine locations and heights of the peaks [57]. Significant peaks were retained if p-values were less than 0.01 based on Poisson distribution.

### Calculation of binding free energy

The minimum binding free energy of each interaction was calculated by RNA hybrid with default settings [58]. Shuffled interactions, shuffled mRNAs and random mRNAs (generated by shuffle function in Perl) served as controls for the comparison of free energy distribution. The median free energy for each profile was calculated using R packages.

### Antagomir treatment

Antagomirs of miR-309, miR-2, and let-7 were purchased from GENEPHARMA (Shanghai, China). Each antagomir was designed as the reverse complement of mature miRNA. Mosquitoes were anesthetized on ice at 12 h PE, and antagomirs were microinjected into the thorax at a dose of 15 pmol. Mosquitoes were then fed with a 10% sucrose solution for 3 days before further analysis.

### Validation of the chimera-defined targets in miR-309 perturbation experiments

The mRNA-seq data were analyzed as previously reported [59]. The log_2_ fold-changes (ant-miR-309/control) were used to perform cumulative distribution function (CDF) analysis to compare the transcripts bearing the chimera-defined miR-309 target sites with those non-target transcripts. The CDFs were also plotted for miR-309 sites overlapping with Ago1 CLIP peaks.

To obtain ribosome footprints, Ribo-seq libraries were constructed using an ARTseq/TruSeq Ribo Profile Kit (Illumina). Briefly, 12-hour old adult female mosquitoes were injected with either antagomiR-309 or control antagomir. Ovaries were dissected at 24 h PBM from 10 injected mosquitoes for each replicate, and were then pulverized in polysome buffer with cycloheximide (0.1 μg/ml) on ice. Ribosome-protected mRNA fragments were purified from Trueseq nuclease-treated lysate using MicroSpin S-400 (GE Life Sciences). Ribosome RNAs were depleted using RiboMinus Kit (Invitrogen). Sample barcodes were incorporated into libraries by 7 cycles of PCR amplification. The libraries were gel-purified and pooled for a single-end 50 bp sequencing. In parallel, total mRNAs were extracted from an aliquot of the ovarian samples to construct cDNA libraries using NEBNext mRNA Library Prep Kit for Illumina sequencing.

Reads from Ribo-seq were processed to remove contaminating rRNA and tRNA using Bowtie. Changes were made to default parameter in removing rRNA reads: maximum mismatches allowed in seed (-n = 2), seed length (-l = 23) and maximum reported alignments (-k = 1). Bowtie was set to perform end-to-end alignment without mismatch to remove tRNA. Cleaned reads were mapped to the *An*. *gambiae* genome (AgamP4.4) using TopHat without looking for novel junctions. After mapping, read counts were calculated with HTSeq set [60] and analyzed with R package DEseq2 to perform ribosome association analysis. Translational efficiency was calculated by normalizing ribosome association against their mRNA levels utilizing RiboDiff [61].

### Luciferase reporter assay

HEK293T cell line was grown in DMEM media (Gibco, Life Technologies) containing 10% (vol/vol) heat-inactivated FBS (Atlanta Biologicals) at 37°C in a humidified incubator supplied with 5% CO_2_. The psiCheck-2 reporters were constructed by inserting the 3′-UTR fragments that harbor putative miR-309 target sites into the psiCheck-2 vector (Promega). Thereafter, 100 ng of psiCheck-2 reporters with 100 nM of synthetic aae-miR-309 miScript miRNA Mimic (Qiagen) or AllStars Negative Control siRNA (Qiagen) were cotransfected into HEK293T cells by using FuGENE HD Transfection Reagent (Promega). Cells were collected and lysed at 48 h after transfection. Luciferase activities were measured using the dual-luciferase reporter assay system (Promega). Each sample was performed in triplicate and transfections were repeated three times.

### Motif analysis

Overrepresented motifs were identified in chimeric reads using MEME with the following settings: -dna -mod zoops -maxw 7 -nmotifs 1. Motifs identified in targets of each miRNA were then aligned to the reverse-complemented miRNA sequence using FIMO [62], with the setting-output-pthresh 0.01. Fifty-six high-confidence motifs were generated with FIMO q-value (FDR) < 0.05, and MEME Bonferroni-corrected p-value < 0.05. We used Homer to perform *de novo* motif usage analysis across the time course. Only miRNAs with more than 30 unique chimeras-defined sites were considered. Background sequences totally five times the number of target sequences were randomly selected from other miRNA chimeras. The retained motifs were subsequently filtered (match score ≥ 0.3, information content per bp ≥ 1.5 and confidence ≥ 1.0). Heatmap showing the percentage of motif presence was created using the R pheatmap package.

### Dynamics of miRNA-mRNA interactions

To analyze the temporal profile of miRNA-mRNA interactions, chimeras-supported Ago1 binding peaks were normalized to mRNA levels at individual time points as previously described [63]. RNA abundance was determined by RNA-seq in this study. Normalized data were loaded into Gene Expression Dynamics Inspector (GEDI v2.1) with the default parameter (26×25 grids) to perform dynamic analysis [64] and visualize pattern changes during adult reproduction. GEDI employs the SOM algorithm to assign interactions with similar trends into close tiles. The Z-score of individual interaction was plotted in R to validate the clustering. Metabolic interactions were classified based on functional annotation of *An*. *gambiae* genes using the eggNOG database (v3.0).

### GO and KEGG enrichment analysis

The selected gene ID list was taken as an input for functional enrichment analysis with DAVID [65]. The top 5 ranked GO terms were chosen. Enrichment score was calculated as -log_10_(p-value).

## Supporting information

S1 Fig Experimental procedure of CLEAR-CLIP. (A) Scheme of experiment. Tissue lysates were prepared from UV cross-linked mosquito abdomens. Endogenous *An*. *gambiae* Ago1 was immunopurified and washed stringently. The RNA ends in the Ago1-RNA complexes were treated with T4 Polynucleotide Kinase and ligated together. RNAs associated with Ago1 were then recovered from an SDS-PAGE gel for library construction and sequencing. (B) Autoradiogram of ^32^P-labelled RNAs cross-linked to Ago1. Ago1 was precipitated by a specific antibody. Rabbit IgG was used as control. (C) Autoradiogram of the Ago1-associated RNA after digestion with various amounts of RNase I. Note that RNA smear disappeared after over digestion by RNase I. (D) PCR products amplified after linker ligation to the RNA extracted from the gel slice shown in (C) (red rectangle).
(PDF)

S2 Fig miRNAs detected in the Ago1-RNA complexes and miRNA-mRNA chimeras. (A and B) Correlation plots of miRNA abundance in the Ago1-associated RNA and the frequency of miRNAs in the chimeras. Pearson’s correlation coefficients are shown for each time point. (C and D) Frequencies of unique chimeras for individual miRNAs at different time points. The top 20 miRNAs were ranked in descending order.
(PDF)

S3 Fig Characterization of the putative targets of miR-309. (A) The levels of miR-309 were measured in the ovaries by quantitative RT-PCR at 24 h PBM in mosquitoes injected with antagomir-309 or control. Non-inj, uninjected mosquitoes; Ant-NC, injection with control antagomir; Ant-miR-309, injection with antagomir-309. (B) mRNA levels of *SIX4* at 24 h PBM in the mosquito ovaries after injection of antagomir-309. The results from qRT-PCR were analyzed using the two-tailed t-test. *, *p*<0.05. (C) The expression of *SIX4* was measured by mRNA-seq. The error bars and statistical tests were determined using Cuffdiff. FPKM, fragments per kilobase of transcript per million fragments mapped. (D) The pairing between miR-309 and its chimeras-defined targets that were used in the luciferase reporter assays. The pairing was generated using RNAhybrid (v2.1.2). Seed sequences of miRNAs are labeled in red.
(PDF)

S4 Fig Regulation of *kr-h1* by let-7 and miR-2 in luciferase reporter assays. DNA fragments bearing putative binding sites (BS) of let-7 (A) and miR-2 (B) in *kr-h1* were cloned separately into psiCHECK-2. Point mutations, highlighted in bold black letters, were introduced to generate the mutant binding sites. Luciferase reporter assays were performed as described in Fig 3C. Results are expressed as the ratio of the Renilla luciferase activity to the firefly luciferase activity (mean ± SD, n = 3). Statistical analyses were performed using a Student’s t-test (*, *p*<0.05; **, *p*<0.01).
(PDF)

S5 Fig The levels of let-7 and miR-2 were reduced in adult female mosquitoes after injection of antagomirs. Specific (Ant-let-7, Ant-miR-2) and control (Ant-NC) antagomirs were injected into adult female mosquitoes at 12 h PE. The relative amounts of *kr-h1* mRNA, let-7, and miR-2 were measured using qRT-PCR.
(PDF)

S6 Fig *kr-h1* was downregulated by the let-7 mimic in adult mosquitoes. Specific miRNA mimics and control mimic (NC mimic) were injected into adult female mosquitoes at 12 h PE. (A) Western blotting was performed after antagomir treatment to measure the Kr-h1 proteins at 120 h PE (before taking a blood meal) and at 24 h PBM. Relative protein abundance was determined by calculating the ratio of Kr-h1 to β-actin and was then normalized relative to uninjected mosquitoes. Non-inj, the uninjected. (B) Oocyte development in the mimic-treated mosquitoes at 24 h PBM.
(PDF)

S7 Fig Conserved motifs in the targets of *An*. *gambiae* miRNAs. The analysis only included miRNAs that had over 50 unique interactions with various mRNAs altogether in the five time points. Motifs identified by MEME were aligned to the reverse-complemented miRNA sequence using FIMO with the setting–output-pthresh 0.01.
(PDF)

S8 Fig Identified targets of miR-9a-5p, miR-14-3p, and miR-276-3p at different time points. The Venn diagrams show largely different targets of individual miRNAs at each stage during adult reproduction.
(PDF)

S9 Fig Functional annotation of miRNA targets at specified time points. The functional categories of target genes were determined using the eggNOG database (v3.0).
(PDF)

S10 Fig Stage-specific motif usages of miR-14-3p and miR-276-3p. Individual motifs in the targets of miR-14-3p (A) and miR-276-3p (B) at various stages.
(PDF)

S11 Fig Comparison of the miRNA-target interactions in *Anopheles gambiae* and *Aedes aegypti*. Ago1 CLIP analysis of the fat body of adult female *Ae*. *aegypti* has been previously reported by Zhang et al. (2017). Orthologous genes that were mapped with Ago1 CLIP peaks at the specified time points were compared between *An*. *gambiae* and *Ae*. *aegypti*. The *Aedes* Ago CLIP data were filtered to only include peak counts that were more than 5.
(PDF)

S12 Fig Functional annotation of the chimeras-defined targets of selected mosquito miRNAs. The top 5 enriched GO terms and KEGG pathways are shown for the targets of individual miRNAs in *An*. *gambiae*. Functional terms that have been reported in previous studies of *Ae*. *aegypti* or *An*. *gambiae* are highlighted in red.
(PDF)

S13 Fig Regulation of lipid metabolism by miR-8 and miR-14. (A) Levels of triglycerides in female mosquitoes at 120 h PE. Adult female mosquitoes were injected with miRNA mimics shortly after eclosion. Triglycerides were measured calorimetrically (n = 6, with six mosquitoes per sample). Statistical analyses were performed using a Student’s t-test (*, *p*<0.05; **, *p*<0.01). Non-inj, uninjected mosquitoes. (B) Lipid droplets in the fat body. Mosquitoes injected with miRNA mimics were dissected at 120 h PE and the lipid droplets were detected after Nile red staining.
(PDF)

S14 Fig mRNA profiles of key genes involved in carbohydrate and lipid metabolic pathways in adult female *An*. *gambiae*. mRNA levels of the selected genes were measured using qRT-PCR in the fat body at the indicated time points. The heat maps show the qRT-PCR-based expression patterns of those genes during the previtellogenic and vitellogenic phases.
(PDF)

S1 Table Read statistics of CLEAR-CLIP with the omission of T4 RNA ligase 1. (PDF)

S2 Table Read statistics of CLEAR-CLIP with spiked-in bacterial RNA. (PDF)

S3 Table Read statistics for CLEAR-CLIP RNA-Seq libraries. (PDF)

S4 Table Chimeras and Ago1 CLIP Peaks. The heights of Ago1 CLIP peaks were normalized to mRNA abundance. miRNA targets were mapped to the transcriptome assembly that we generated in this study using RNA-seq data from the *Anopheles gambiae* samples (DDBJ/ENA/GenBank accession GIBN00000000).
(XLSX)

S5 Table Primers used in this study. (PDF)

S6 Table Description of metabolic genes showing distinct patterns of interactions with miRNAs. (PDF)

S1 Appendix CLEAR-CLIP Protocol for *An*. *gambiae* Mosquitoes. (PDF)
