# Supplementary figures and images for: Dynamic miRNA-mRNA interactions coordinate gene expression in adult Anopheles gambiae
Source: PLoS Genet. 2020 Apr 27;16(4):e1008765. doi: 10.1371/journal.pgen.1008765 (PMC7205314; doi:10.1371/journal.pgen.1008765)

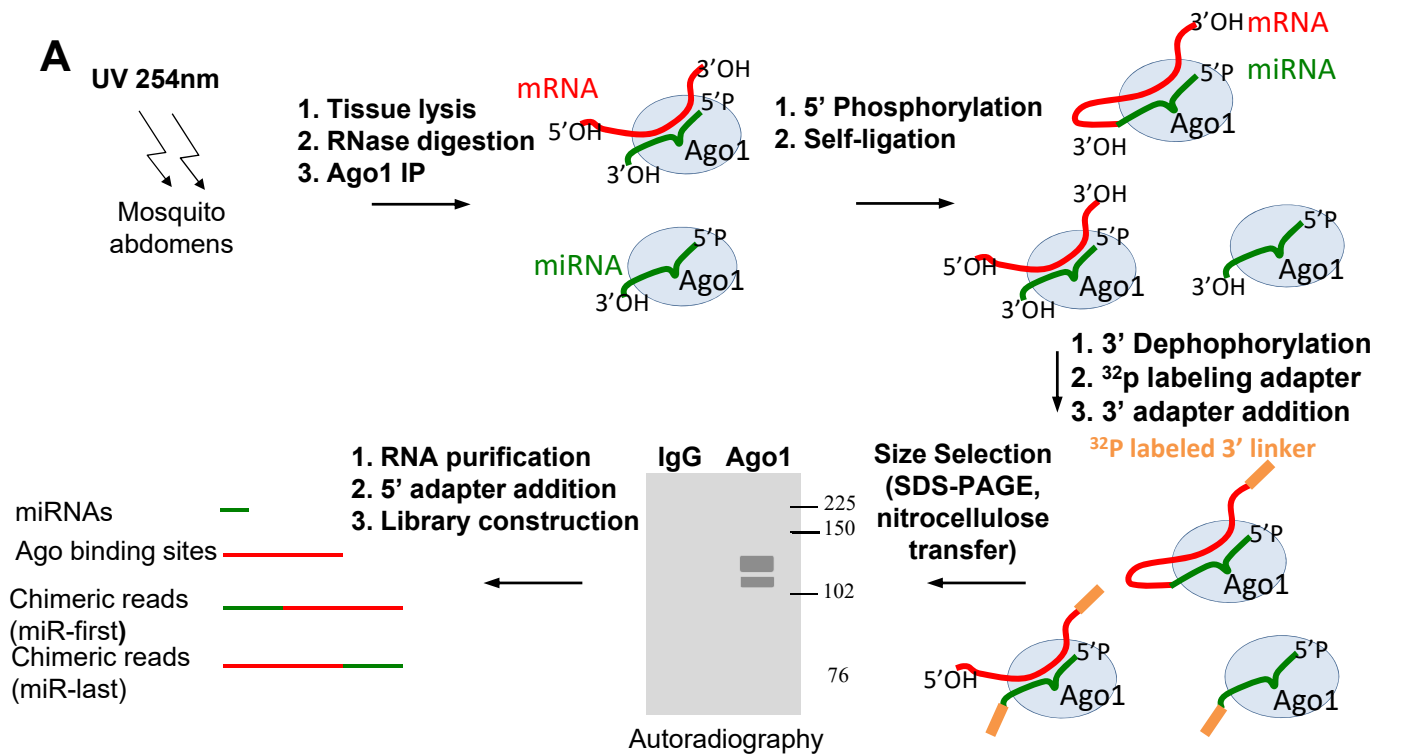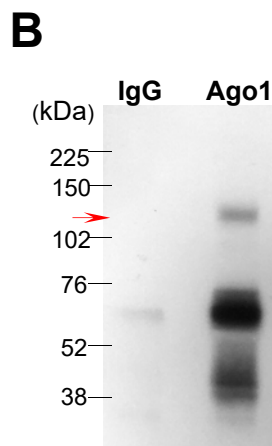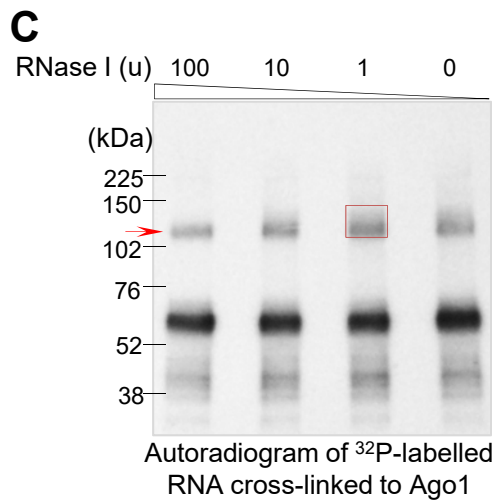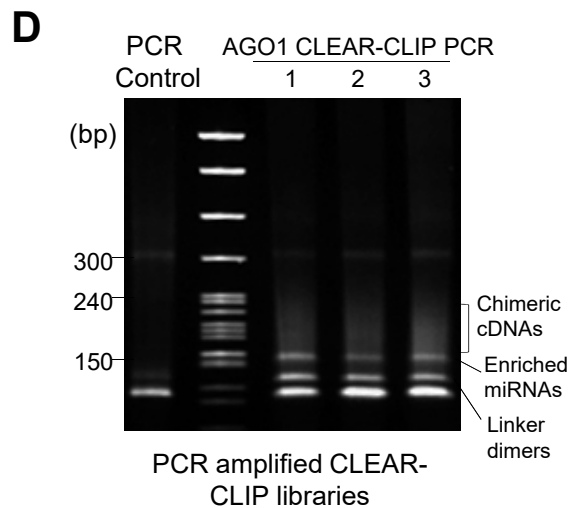

Supplement: S1 Fig — (A) Scheme of experiment. Tissue lysates were prepared from UV cross-linked mosquito abdomens. Endogenous An. gambiae Ago1 was immunopurified and washed stringently. The RNA ends in the Ago1-RNA complexes were treated with T4 Polynucleotide Kinase and ligated together. RNAs associated with Ago1 were then recovered from an SDS-PAGE gel for library construction and sequencing. (B) Autoradiogram of 32P-labelled RNAs cross-linked to Ago1. Ago1 was precipitated by a specific antibody. Rabbit IgG was used as control. (C) Autoradiogram of the Ago1-associated RNA after digestion with various amounts of RNase I. Note that RNA smear disappeared after over digestion by RNase I. (D) PCR products amplified after linker ligation to the RNA extracted from the gel slice shown in (C) (red rectangle). (PDF) [file pgen.1008765.s001.pdf]

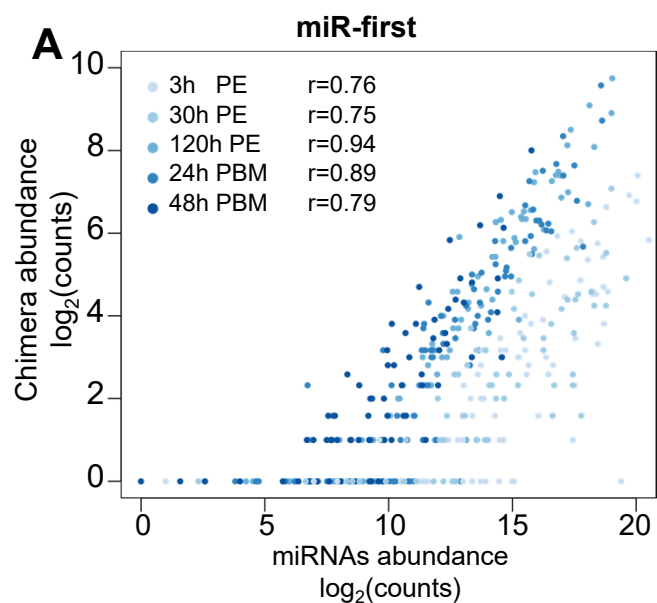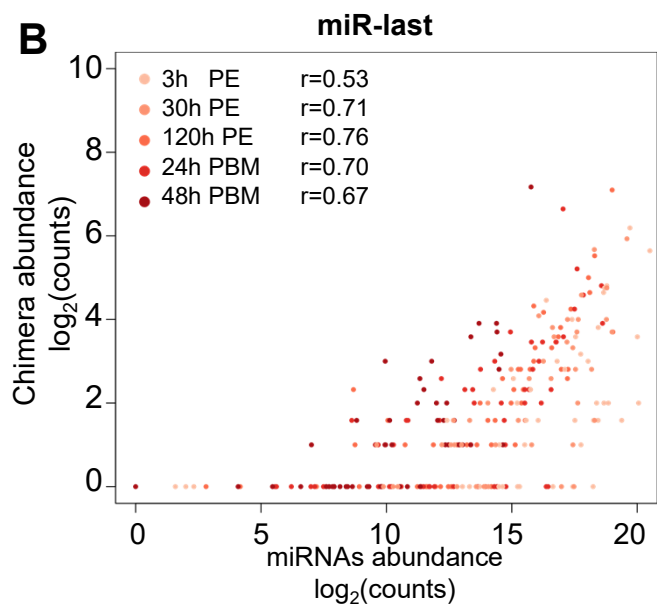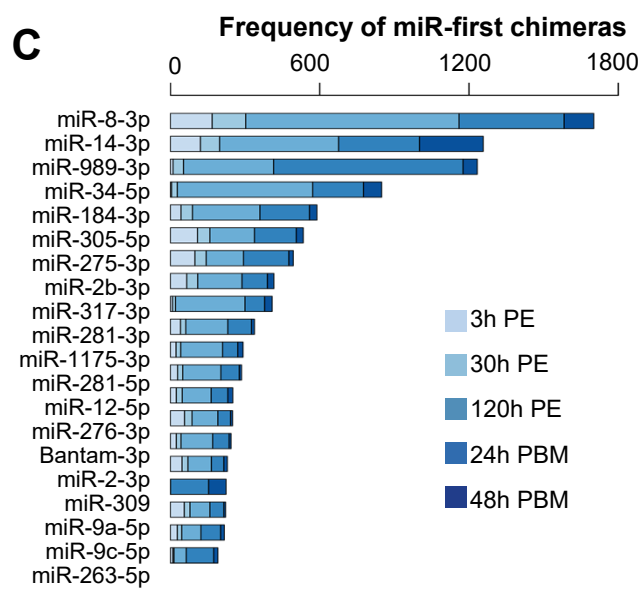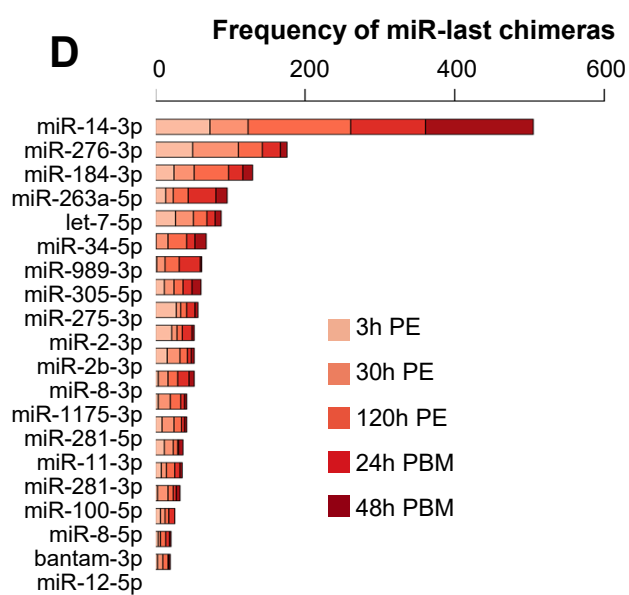

Supplement: S2 Fig — (A and B) Correlation plots of miRNA abundance in the Ago1-associated RNA and the frequency of miRNAs in the chimeras. Pearson’s correlation coefficients are shown for each time point. (C and D) Frequencies of unique chimeras for individual miRNAs at different time points. The top 20 miRNAs were ranked in descending order. (PDF) [file pgen.1008765.s002.pdf]

# A

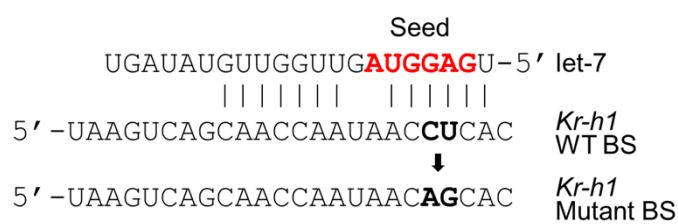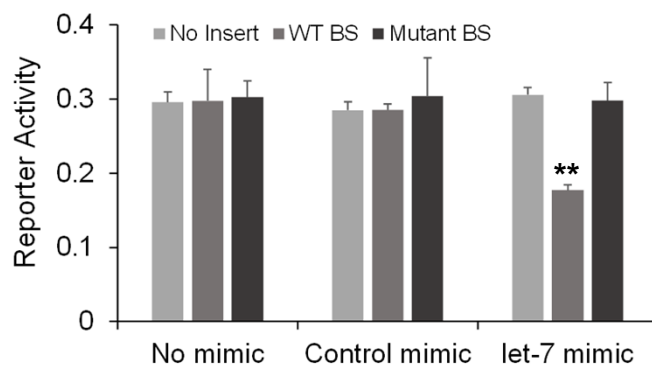

# B

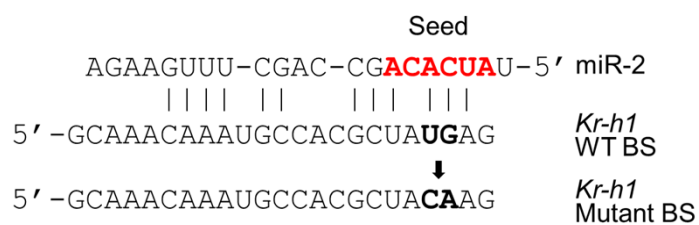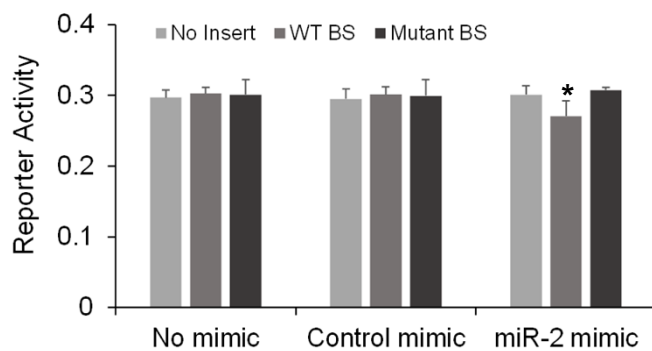

Supplement: S4 Fig — DNA fragments bearing putative binding sites (BS) of let-7 (A) and miR-2 (B) in kr-h1 were cloned separately into psiCHECK-2. Point mutations, highlighted in bold black letters, were introduced to generate the mutant binding sites. Luciferase reporter assays were performed as described in Fig 3C. Results are expressed as the ratio of the Renilla luciferase activity to the firefly luciferase activity (mean ± SD, n = 3). Statistical analyses were performed using a Student’s t-test (*, p<0.05; **, p<0.01). (PDF) [file pgen.1008765.s004.pdf]

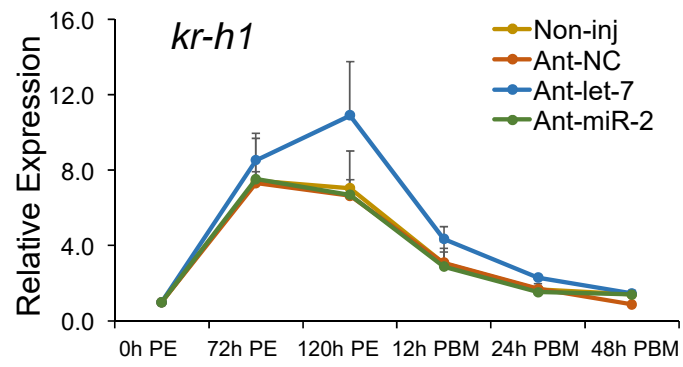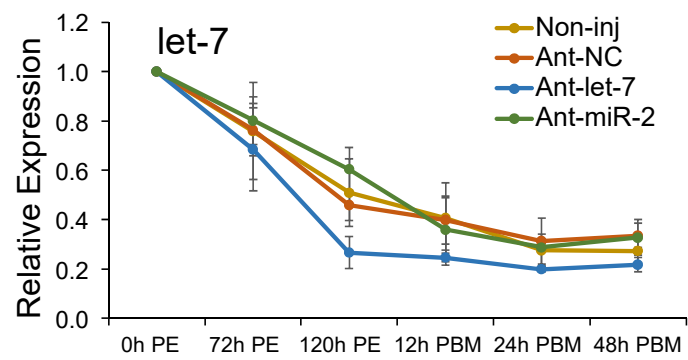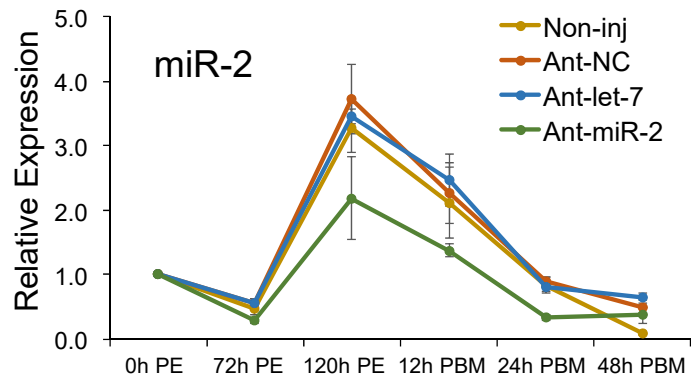

Supplement: S5 Fig — Specific (Ant-let-7, Ant-miR-2) and control (Ant-NC) antagomirs were injected into adult female mosquitoes at 12 h PE. The relative amounts of kr-h1 mRNA, let-7, and miR-2 were measured using qRT-PCR. (PDF) [file pgen.1008765.s005.pdf]

**A**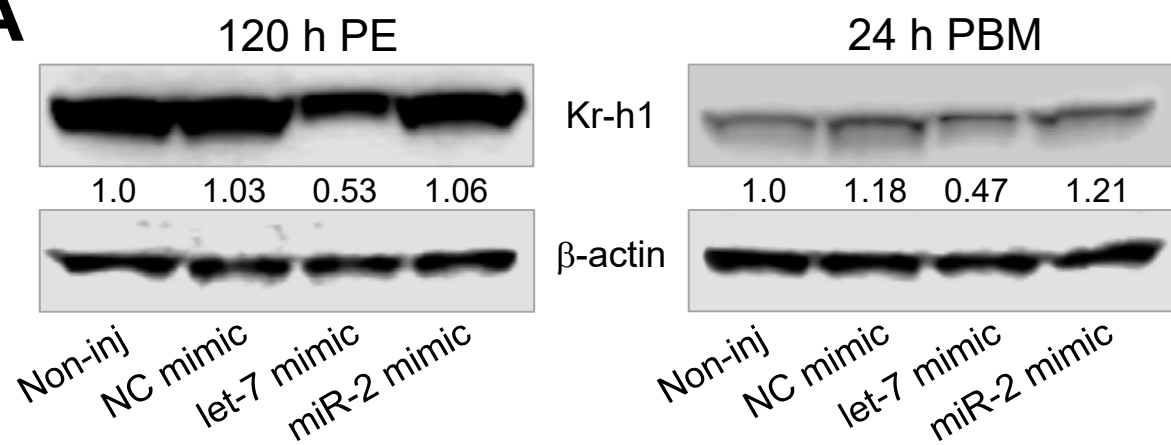**B**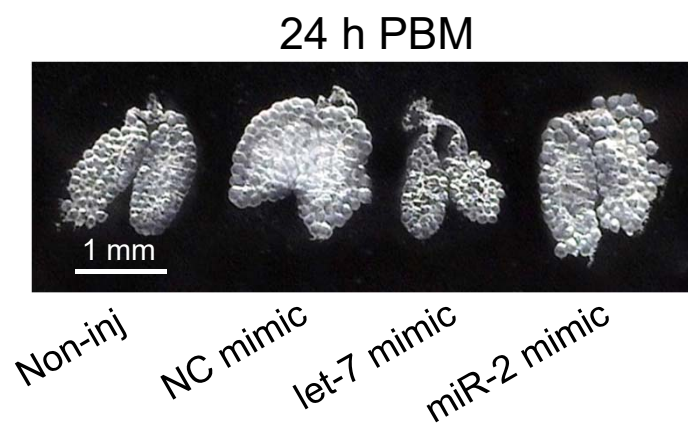

Supplement: S6 Fig — Specific miRNA mimics and control mimic (NC mimic) were injected into adult female mosquitoes at 12 h PE. (A) Western blotting was performed after antagomir treatment to measure the Kr-h1 proteins at 120 h PE (before taking a blood meal) and at 24 h PBM. Relative protein abundance was determined by calculating the ratio of Kr-h1 to β-actin and was then normalized relative to uninjected mosquitoes. Non-inj, the uninjected. (B) Oocyte development in the mimic-treated mosquitoes at 24 h PBM. (PDF) [file pgen.1008765.s006.pdf]

# 56 miRNAs with motif matches

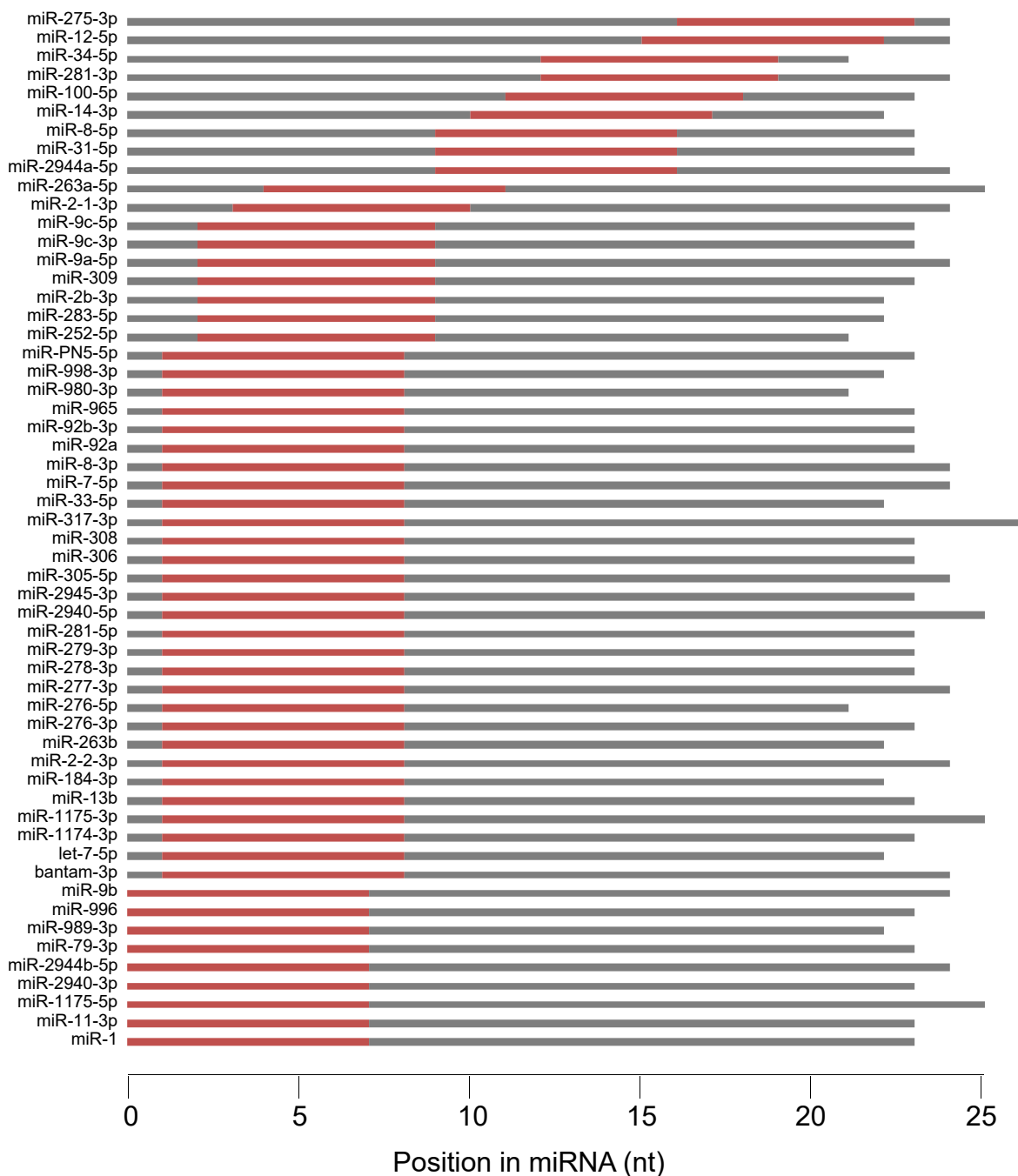

Supplement: S7 Fig — The analysis only included miRNAs that had over 50 unique interactions with various mRNAs altogether in the five time points. Motifs identified by MEME were aligned to the reverse-complemented miRNA sequence using FIMO with the setting–output-pthresh 0.01. (PDF) [file pgen.1008765.s007.pdf]

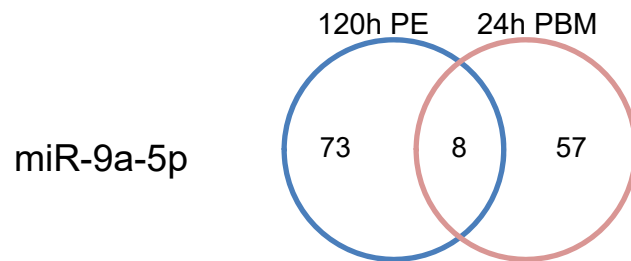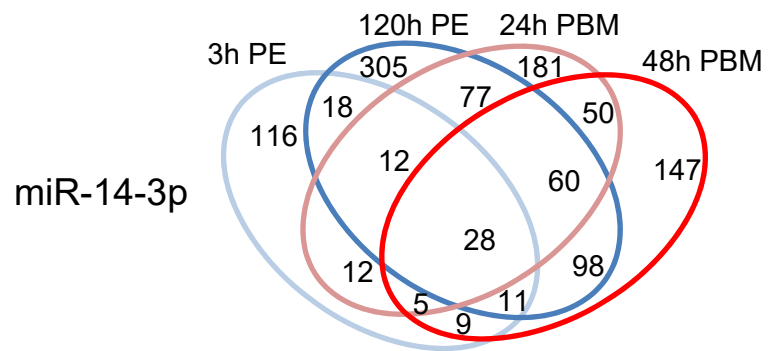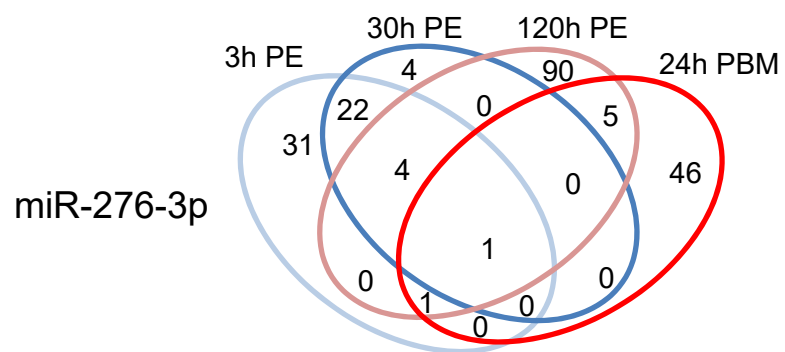

Supplement: S8 Fig — The Venn diagrams show largely different targets of individual miRNAs at each stage during adult reproduction. (PDF) [file pgen.1008765.s008.pdf]

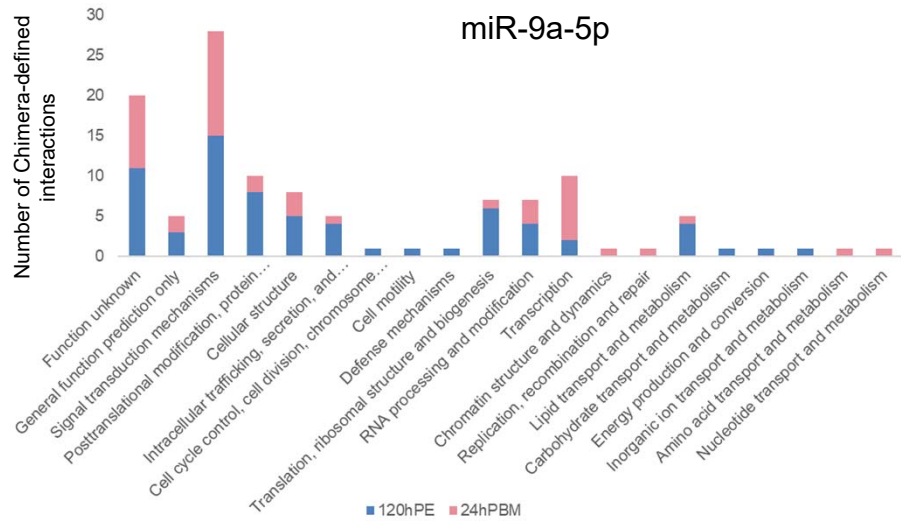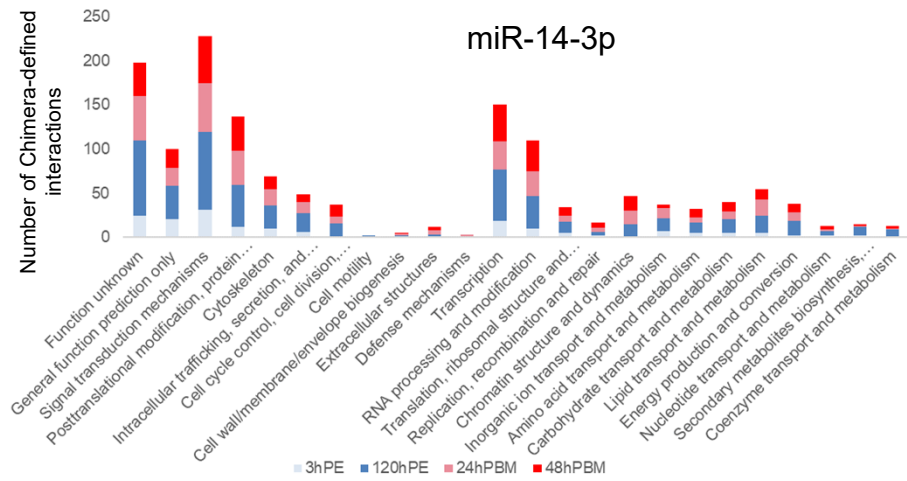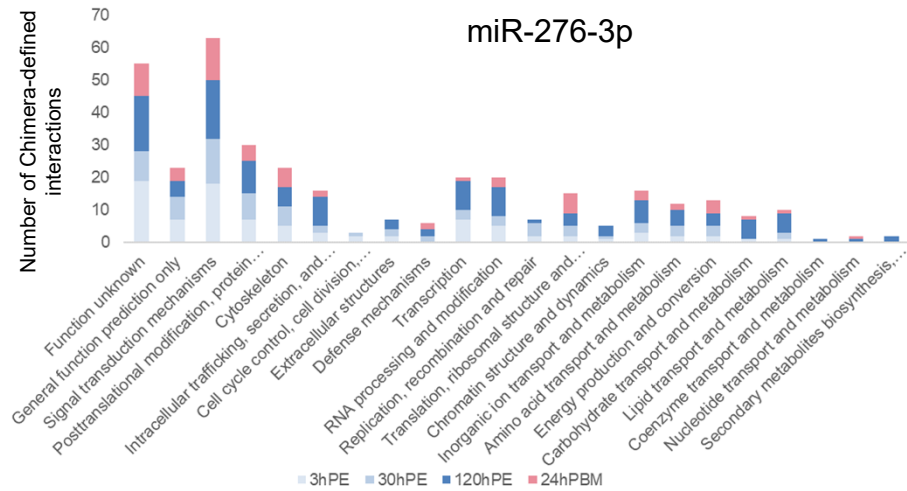

Supplement: S9 Fig — The functional categories of target genes were determined using the eggNOG database (v3.0). (PDF) [file pgen.1008765.s009.pdf]

KEGG pathway

GO biological process

### miR-8-3p

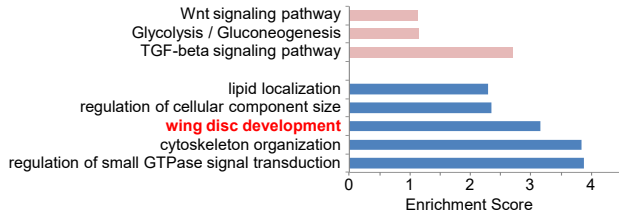

### miR-277-3p

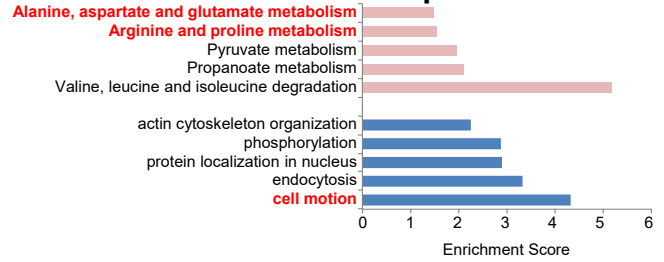

### miR-1890-3p

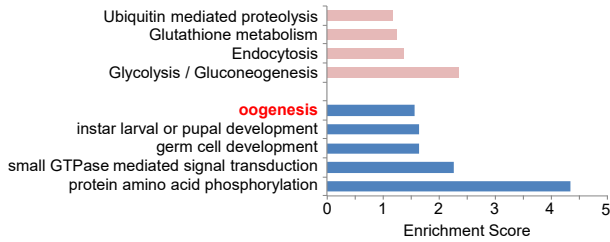

### miR-1174-3p

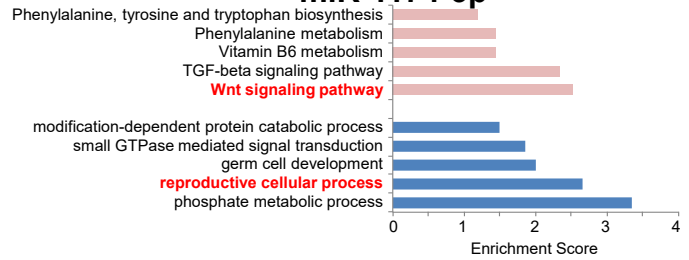

### miR-309

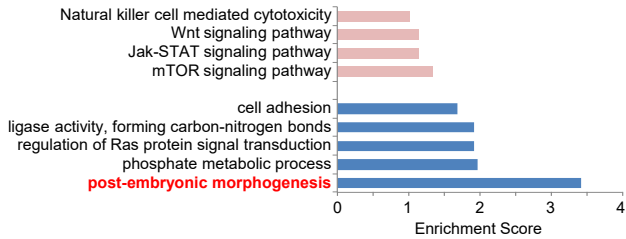

### miR-305-5p

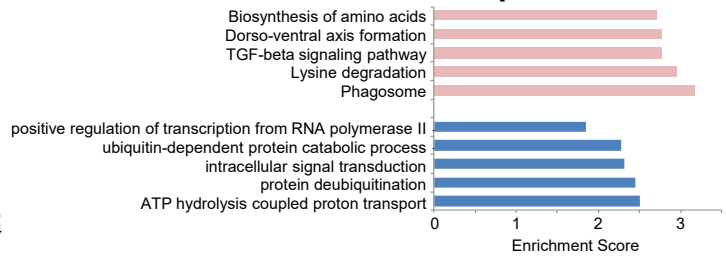

### miR-14

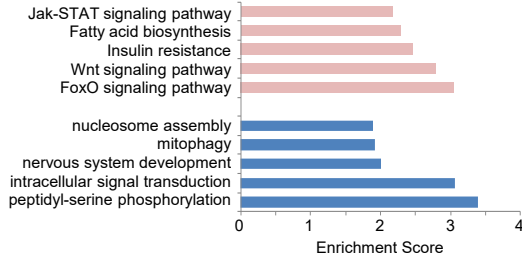

### miR-34-5p

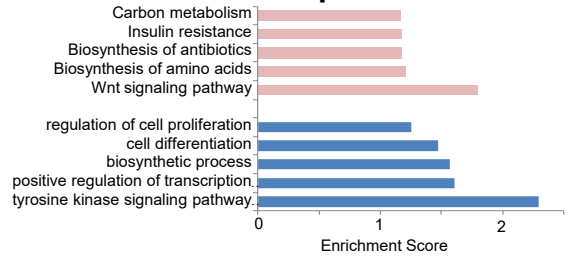

### miR-184

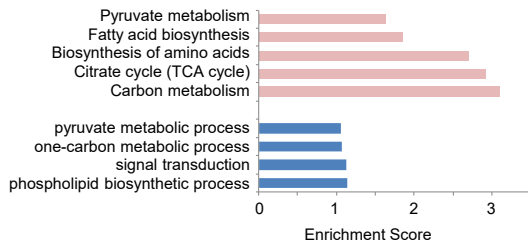

### miR-989

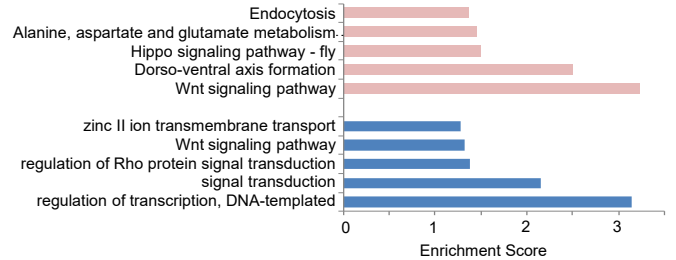

Supplement: S12 Fig — The top 5 enriched GO terms and KEGG pathways are shown for the targets of individual miRNAs in An. gambiae. Functional terms that have been reported in previous studies of Ae. aegypti or An. gambiae are highlighted in red. (PDF) [file pgen.1008765.s012.pdf]

**A**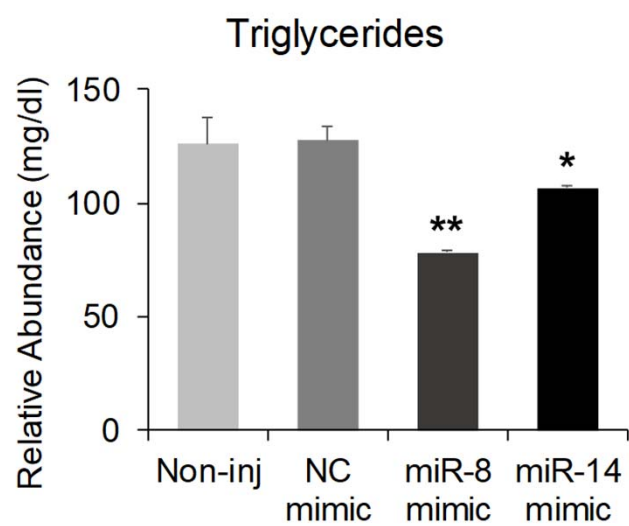**B**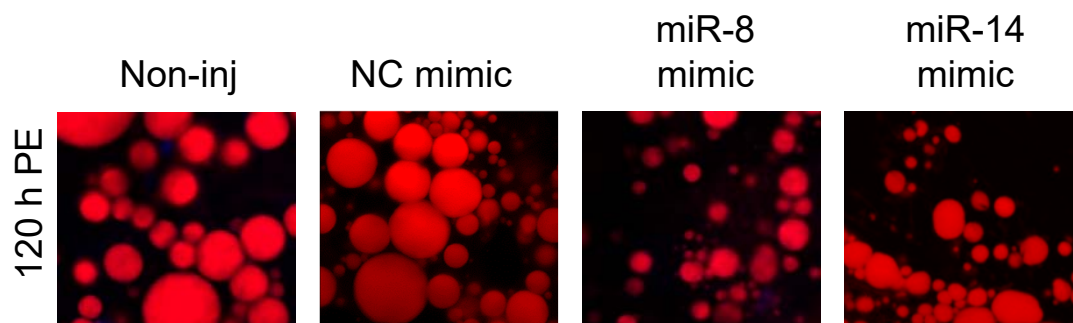

Supplement: S13 Fig — (A) Levels of triglycerides in female mosquitoes at 120 h PE. Adult female mosquitoes were injected with miRNA mimics shortly after eclosion. Triglycerides were measured calorimetrically (n = 6, with six mosquitoes per sample). Statistical analyses were performed using a Student’s t-test (*, p<0.05; **, p<0.01). Non-inj, uninjected mosquitoes. (B) Lipid droplets in the fat body. Mosquitoes injected with miRNA mimics were dissected at 120 h PE and the lipid droplets were detected after Nile red staining. (PDF) [file pgen.1008765.s013.pdf]

## Carbohydrate Metabolic Genes

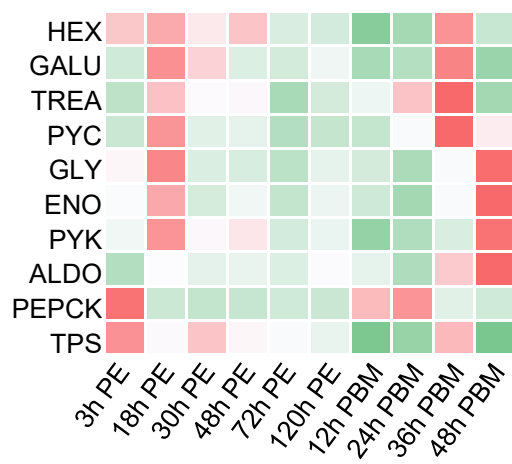

## Lipid Metabolic Genes

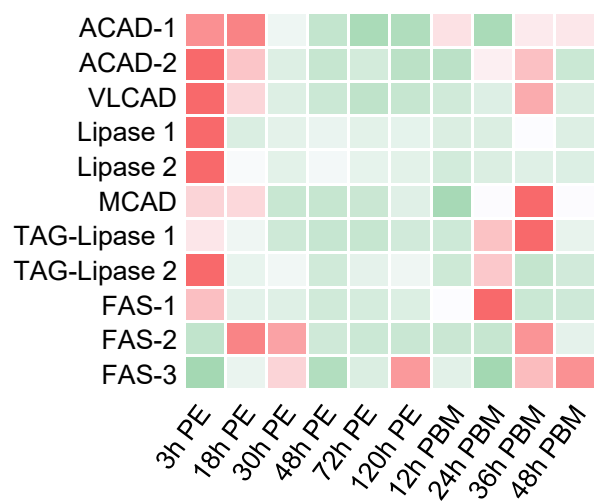

-2 -1 0 1 2

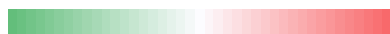

Z-normalized fold-change

Supplement: S14 Fig — mRNA levels of the selected genes were measured using qRT-PCR in the fat body at the indicated time points. The heat maps show the qRT-PCR-based expression patterns of those genes during the previtellogenic and vitellogenic phases. (PDF) [file pgen.1008765.s014.pdf]
