# Supplementary material for: Dynamic miRNA-mRNA interactions coordinate gene expression in adult Anopheles gambiae
Source: PLoS Genet. 2020 Apr 27;16(4):e1008765. doi: 10.1371/journal.pgen.1008765 (PMC7205314; doi:10.1371/journal.pgen.1008765)
Supplement: S3 Fig — (A) The levels of miR-309 were measured in the ovaries by quantitative RT-PCR at 24 h PBM in mosquitoes injected with antagomir-309 or control. Non-inj, uninjected mosquitoes; Ant-NC, injection with control antagomir; Ant-miR-309, injection with antagomir-309. (B) mRNA levels of SIX4 at 24 h PBM in the mosquito ovaries after injection of antagomir-309. The results from qRT-PCR were analyzed using the two-tailed t-test. *, p<0.05. (C) The expression of SIX4 was measured by mRNA-seq. The error bars and statistical tests were determined using Cuffdiff. FPKM, fragments per kilobase of transcript per million fragments mapped. (D) The pairing between miR-309 and its chimeras-defined targets that were used in the luciferase reporter assays. The pairing was generated using RNAhybrid (v2.1.2). Seed sequences of miRNAs are labeled in red. (PDF) [file pgen.1008765.s003.pdf]

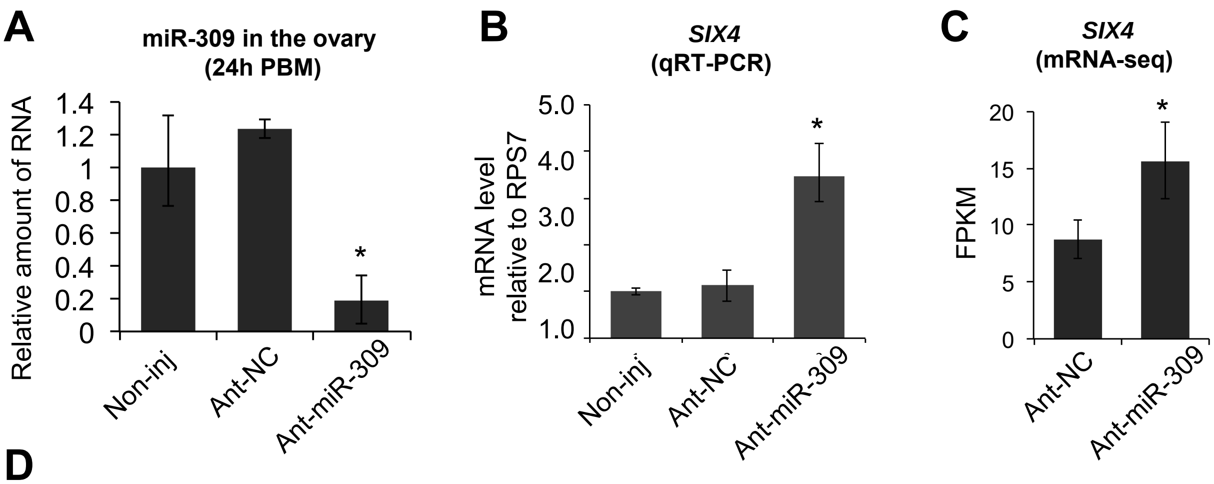

D

Canonical Seed-paired match (2-7)

```
>miR-309_AGAP007538-RA_5UTR (-24.4 kcal/mol)
target 5' C          A          C 3'
           UGCGAC AGC    UCCAGUG
           ACGCUG UUG    GGGUCAC
miRNA  3'       U   AAAC        U 5'

>miR-309_AGAP006569-RA_5UTR (-22.7 kcal/mol)
target 5'   A      G A          U 3'
             GGCAAA U    CCAGUGA
             CUGUUU A    GGUCACU
miRNA  3' ACG      G AACG          5'

>miR-309_AGAP000007-RA_CDS (-20.4 kcal/mol)
target 5'   G     CG  A    A      C 3'
             GACAA  GC  UGU CCAGUG
             CUGUU  UG  ACG GGUCAC
miRNA  3' ACG         AA          U 5'

>miR-309_AGAP005555-RA_CDS (-25.1 kcal/mol)
target 5' G    G    CGU        U 3'
           GCGA AAAC    UCCAGUG
           CGCU UUUG    GGGUCAC
miRNA  3' A    G    AAAC       U 5'

>miR-309_AGAP011700-RA_3UTR (-20.4 kcal/mol)
target 5' C  CA     ACCCUC      U 3'
           GC   AAAC      CCCAGUG
           CG   UUUG      GGGUCAC
miRNA  3' A  CUG    AAAC        U 5'

>miR-309_AGAP011067-RA_3UTR (-27.1 kcal/mol)
target 5'   A       GUAA         A 3'
             GACAAAC    UG CCAGUGA
             CUGUUUG    AC GGUCACU
miRNA  3' ACG       AA    G         5'
```

Offset seed-paired match (3-8)

```
>miR-309_AGAP007623-RA_CDS (-24.7 kcal/mol)
target 5' G      CUGAAAUUU          U  3'
           GCGACG         CUUU CCCAGU
           CGCUGU         GAAA GGGUCA
miRNA  3' A      UU            C      CU 5'

>miR-309_AGAP007815-RA_CDS (-26.4 kcal/mol)
target 5' U       C   GUAA  A     C  3'
           UGCGACA AAU    UG CCCAGU
           ACGCUGU UUG    AC GGGUCA
miRNA  3'             AA          CU 5'
```

Bulged seed-paired match

```
>miR-309_AGAP009132-RB_3UTR (-29.0 kcal/mol)
target 5' U        CAGUAC     U  U     U  3'
           UGCGACAA      GCUUU GC CCAGU
           ACGCUGUU      UGAAA CG GGUCA
miRNA  3'                               CU 5'

>miR-309_AGAP011336-RA_3UTR (-26.5 kcal/mol)
target 5'    C      GAGAG    G     A 3'
              ACAAAC     UGCC AGUGA
              UGUUUG     ACGG UCACU
miRNA  3' ACGC      AA       G       5'
```
