# Supplementary material for: Dynamic miRNA-mRNA interactions coordinate gene expression in adult Anopheles gambiae
Source: PLoS Genet. 2020 Apr 27;16(4):e1008765. doi: 10.1371/journal.pgen.1008765 (PMC7205314; doi:10.1371/journal.pgen.1008765)
Supplement: S10 Fig — Individual motifs in the targets of miR-14-3p (A) and miR-276-3p (B) at various stages. (PDF) [file pgen.1008765.s010.pdf]

A

Motifs in the targets of miR-14-3p

Stages	Enriched motifs complementary to miRNA	Number of targets possessing the motif	Portion of targets possessing the motif
3h PE	nts. 2-8	13	10.7%
	nts. 12-18	61	50.4%
	nts. 2-8 + nts.12-18	12	9.9%
120h PE	nts. 2-8	31	6.6%
	nts. 11-17	195	41.3%
	nts. 2-8 + nts. 11-17	6	1.3%
24h PBM	nts. 3-9	24	7.3%
	nts. 11-17	146	44.4%
	nts. 3-9 + nts. 11-17	12	3.7%
48h PBM	nts. 11-17	68	26.8%
	nts. 13-19	69	27.2%
	nts. 11-17 + nts. 13-19	64	25.2%

B

Motifs in the targets of miR-276-3p

Stages	Enriched motifs complementary to miRNA	Number of targets possessing the motif	Portion of targets possessing the motif
3h PE	nts. 1-7	29	50.9%
30h PE	nts. 15-21	14	46.7%
120h PE	nts. 2-8	15	14.4%
	nts. 14-20	28	26.9%
	nts. 2-8 + nts. 14-20	5	4.8%
24h PBM	nts. 1-7	15	29.4%
	nts. 16-22	13	25.5%
	nts. 1-7 + nts. 16-22	8	15.7%
