# Supplementary material for: Dynamic miRNA-mRNA interactions coordinate gene expression in adult Anopheles gambiae
Source: PLoS Genet. 2020 Apr 27;16(4):e1008765. doi: 10.1371/journal.pgen.1008765 (PMC7205314; doi:10.1371/journal.pgen.1008765)
Supplement: S11 Fig — Ago1 CLIP analysis of the fat body of adult female Ae. aegypti has been previously reported by Zhang et al. (2017). Orthologous genes that were mapped with Ago1 CLIP peaks at the specified time points were compared between An. gambiae and Ae. aegypti. The Aedes Ago CLIP data were filtered to only include peak counts that were more than 5. (PDF) [file pgen.1008765.s011.pdf]

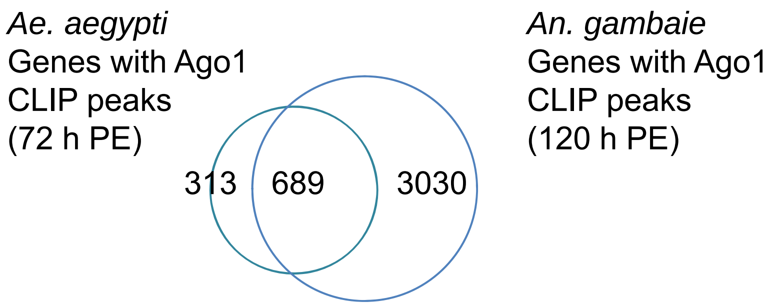
Ae. aegypti
Genes with Ago1
CLIP peaks
(72 h PE)
An. gambaie
Genes with Ago1
CLIP peaks
(120 h PE)
313
689
3030

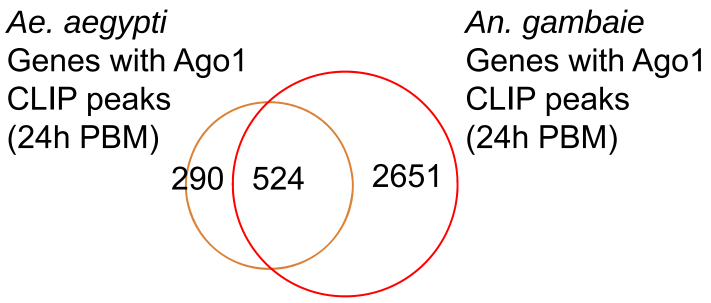
Ae. aegypti
Genes with Ago1
CLIP peaks
(24h PBM)
An. gambaie
Genes with Ago1
CLIP peaks
(24h PBM)
290
524
2651
