# Supplementary material for: Dynamic miRNA-mRNA interactions coordinate gene expression in adult Anopheles gambiae
Source: PLoS Genet. 2020 Apr 27;16(4):e1008765. doi: 10.1371/journal.pgen.1008765 (PMC7205314; doi:10.1371/journal.pgen.1008765)
Supplement: S1 Table — (PDF) [file pgen.1008765.s015.pdf]

S1 Table. Read statistics of CLEAR-CLIP with the omission of T4 RNA ligase 1

Sample	Time point	Condition	Unique reads, mapped to *An. gambiae*	Unique miR-first chimeras	Unique miR-last chimeras	Fraction of chimeric reads
Mosquito	24 h PBM	T4 RNA ligase 1	1076718	22262	2162	2.27%
Mosquito	24 h PBM	No ligase	1283471	2456	1441	0.30%
