# Supplementary material for: Dynamic miRNA-mRNA interactions coordinate gene expression in adult Anopheles gambiae
Source: PLoS Genet. 2020 Apr 27;16(4):e1008765. doi: 10.1371/journal.pgen.1008765 (PMC7205314; doi:10.1371/journal.pgen.1008765)
Supplement: S2 Table — (PDF) [file pgen.1008765.s016.pdf]

S2 Table. Read statistics of CLEAR-CLIP with spiked-in bacterial RNA

Sample	Time point	Unique reads, mapped to *An. gambiae*	Unique reads, mapped to *E. coli*	Unique chimeras, mapped to *An. gambiae*	Unique chimeras, mapped to *E. coli*	Fraction of chimeric reads, mapped to *E. coli*
mosquito only	24 h PBM	1076718	3813	24424	579	2.37%
mosquito+*E.coli* (1:1)	24 h PBM	1014652	5870	22219	618	2.78%
