# Supplementary material for: Dynamic miRNA-mRNA interactions coordinate gene expression in adult Anopheles gambiae
Source: PLoS Genet. 2020 Apr 27;16(4):e1008765. doi: 10.1371/journal.pgen.1008765 (PMC7205314; doi:10.1371/journal.pgen.1008765)
Supplement: S3 Table — (PDF) [file pgen.1008765.s017.pdf]

S3 Table. Read statistics for CLEAR-CLIP RNA-Seq libraries

Sample	Replicate	Clean reads	Unique reads	Mapped reads	Chimeric reads	Percent of Chimeric	Unique miR-first	Unique miR-last	Supported by CLIP reads
3 h PE	1	23718508	1958583	917535	10503	1.14%			
3 h PE	2	28530465	1482846	787609	13944	1.77%	1421	473	76.3%
3 h PE	3	20151195	2289820	962104	7701	0.80%			
30 h PE	1	10122615	543755	290875	3961	1.36%			
30 h PE	2	15436457	901142	460160	7079	1.54%	936	488	64.2%
30 h PE	3	11676607	775329	392306	6100	1.55%			
120 h PE	1	17444532	720851	557432	12896	2.31%			
120 h PE	2	14817122	530275	431155	11894	2.76%	6093	592	88.0%
120 h PE	3	17223186	596502	472060	11688	2.48%			
24 h PBM	1	15681837	622612	503618	4601	0.91%			
24 h PBM	2	13792735	517278	402076	4488	1.12%	4484	484	90.4%
24 h PBM	3	14808988	520564	423436	4813	1.14%			
24 h PBM	4	39703332	896123	751460	6381	0.85%			
48 h PBM	1	17245912	748665	593243	1267	0.21%			
48 h PBM	2	16162544	659124	564399	1068	0.19%	1100	360	91.9%
48 h PBM	3	15831523	883009	770028	1210	0.16%			
