# Supplementary material for: Dynamic miRNA-mRNA interactions coordinate gene expression in adult Anopheles gambiae
Source: PLoS Genet. 2020 Apr 27;16(4):e1008765. doi: 10.1371/journal.pgen.1008765 (PMC7205314; doi:10.1371/journal.pgen.1008765)
Supplement: S5 Table — (PDF) [file pgen.1008765.s019.pdf]

S5 Table. Primers used in this study.

Primer	Sequence (5' to 3')	use
R3	AGATCGGAAGAGCACACGTCT/3ddc/	3'linker for CLEAR-CLIP
R5	/5InvddT/GTTCArGrArGrUrUrCrUrArCrArGrUrCrCrGrArCrGrArUrCrNrNrNrUrArArGrC	5'linker for CLEAR-CLIP
RTp	AGACGTGTGCTCTTCCGATCT	RT for CLEAR-CLIP
AGAP000007_F	CCGACTCGAGCAATCGCGACAAGTACACAATAC	primer to clone into psiCheck2 for luciferease assay
AGAP000007_R	GAATGCGGCCGCCATCTTTCTTCCACACAATC	primer to clone into psiCheck2 for luciferease assay
AGAP011067_F	CCGACTCGAGGTAAATAGCGATGGGAGAAGGG	primer to clone into psiCheck2 for luciferease assay
AGAP011067_R	GAATGCGGCCGCTTCTTCATTTCTGTACAGCGTTTG	primer to clone into psiCheck2 for luciferease assay
AGAP011336_F	CCGACTCGAGGAAACGAAACAGCGTGAAAGG	primer to clone into psiCheck2 for luciferease assay
AGAP011336_R	GAATGCGGCCGCCGATAGCAGTCCAACATCAA	primer to clone into psiCheck2 for luciferease assay
AGAP011700_F	CCGACTCGAGGCACACTACACATCCTTCTCTAC	primer to clone into psiCheck2 for luciferease assay
AGAP011700_R	GAATGCGGCCGCTCTCTCTCTTTCTCCCTTCACT	primer to clone into psiCheck2 for luciferease assay
AGAP005555_F	CCGACTCGAGGCAACTACACCAGCAACAAG	primer to clone into psiCheck2 for luciferease assay
AGAP005555_R	GAATGCGGCCGCAAAGCAAACGTCACAGGAG	primer to clone into psiCheck2 for luciferease assay
AGAP006569_F	CCGACTCGAGTGTGTGTGGTGCGATTGT	primer to clone into psiCheck2 for luciferease assay
AGAP006569_R	GAATGCGGCCGCGTGTTTGCTGGGTTTCCTTTC	primer to clone into psiCheck2 for luciferease assay
AGAP007538_F	CCGACTCGAGAAGATAGCATCCGACCGTTAC	primer to clone into psiCheck2 for luciferease assay
AGAP007538_R	GAATGCGGCCGCTGTGTGTTGCCTCCAGTTT	primer to clone into psiCheck2 for luciferease assay
AGAP007623_F	CCGACTCGAGTATTAGTCGACAATGCGCAAAC	primer to clone into psiCheck2 for luciferease assay
AGAP007623_R	GAATGCGGCCGCCTGCGACTTTAGCTCGATG	primer to clone into psiCheck2 for luciferease assay
AGAP007815_F	CCGACTCGAGCACATCTGGTCGGTTGATGTA	primer to clone into psiCheck2 for luciferease assay
AGAP007815_R	GAATGCGGCCGCTGTTGGGCACTAATGGTCT	primer to clone into psiCheck2 for luciferease assay
AGAP009132_F	CCGACTCGAGACTTCACCACAGTCTCCTTAAC	primer to clone into psiCheck2 for luciferease assay
AGAP009132_R	GAATGCGGCCGCACACTAGCTCCTTGTAGCATTC	primer to clone into psiCheck2 for luciferease assay
let-7_krh1_F	CCGACTCGAGCGCAAACAAGTCCTCGTAGT	primer to clone into psiCheck2 for luciferease assay
let-7_krh1_R	GAATGCGGCCGCGTGTGGGTGTGTGAGTGTAT	primer to clone into psiCheck2 for luciferease assay
miR-2_krh1_F	CCGACTCGAGCCATACAAATGCCCGGTAGA	primer to clone into psiCheck2 for luciferease assay
miR-2_krh1_R	GAATGCGGCCGCTCATGCTGGACGAATAGTC	primer to clone into psiCheck2 for luciferease assay
miR-309	TCACTGGGCAAAGTTTGTCGCA	miRNA qRT-PCR
let-7-5p	TGAGGTAGTTGGTTGTATAGT	miRNA qRT-PCR
miR-2	TCACAGCCAGCTTTGATGAGC	miRNA qRT-PCR
universal miR primer	GAATCGAGCACCAGTTACGC	miRNA qRT-PCR
RPS7_F	GAAGAAGTGGCCATCATTCC	qRT-PCR
RPS7_R	CGCAGGCGCTTCATCAGATG	qRT-PCR
SIX4_F	CGGGAAGATCACTGTGTGATAG	qRT-PCR
SIX4_R	CCCTTCTCCCATCGCTATTTAC	qRT-PCR
