# Supplementary material for: Dynamic miRNA-mRNA interactions coordinate gene expression in adult Anopheles gambiae
Source: PLoS Genet. 2020 Apr 27;16(4):e1008765. doi: 10.1371/journal.pgen.1008765 (PMC7205314; doi:10.1371/journal.pgen.1008765)
Supplement: S6 Table — (PDF) [file pgen.1008765.s020.pdf]

S6 Table. Description of metabolic genes showing distinct patterns of interactions with miRNAs

**ACO1**	aconitate hydratase 1 / homoaconitase
**ACSL**	long-chain acyl-CoA synthetase
**AGXT2**	alanine-glyoxylate aminotransferase 2-like
**ALT**	alanine transaminase
**ASL**	argininosuccinate lyase
**BCATM2**	branched-chain-amino-acid aminotransferase 2
**C4ST**	chondroitin 4-sulfotransferase
**COX5B**	Mitochondrial cytochrome c oxidase subunit 5B isoform 1
**CYCS**	cytochrome c
**CYTb5r**	cytochrome b5-related
**ELOVL1**	Elongation of very long chain fatty acids protein 1
**FAS1**	fatty acid synthase 1
**FAS2**	fatty acid synthase 2
**FAS3**	fatty acid synthase 3
**FBA**	fructose biphosphate aldolase
**FHTAC**	F-type H+-transporting ATPase subunit c
**FLVCR1**	MFS transporter, FLVCR family, feline leukemia virus subgroup C receptor-relat
**GHMT**	glycine hydroxymethyltransferase
**GOT2**	aspartate aminotransferase%2C mitochondrial
**GSA**	glucosylceramidase
**Lip3**	lipase 3
**LYZ**	C-Type Lysozyme.
**MAN1A1**	mannosyl-oligosaccharide alpha-1,2-mannosidase
**MCOXVIC**	Mitochondrial cytochrome c oxidase subunit VIC
**NDUFA5**	NADH dehydrogenase (ubiquinone) 1 alpha subcomplex 5
**PEPCK**	phosphoenolpyruvate carboxykinase (GTP)
**PGRPs**	peptidoglycan recognition protein (short)
**PiT**	inorganic phosphate cotransporter
**PK**	pyruvate kinase
**PLPdec**	group ii plp decarboxylase
**Rim2**	Mitochondrial carrier protein (Rim2)
**RLBP1**	cellular retinaldehyde-binding protein
**SCD**	stearoyl-CoA desaturase (delta-9 desaturase)
**SP**	starch phosphorylase
**TDO2**	tryptophan 2,3-dioxygenase
**TH**	tyrosine 3-monooxygenase
**TMEMB**	Trimeric intracellular cation channel type B
**Tret1**	facilitated trehalose transporter Tret1
**VHAB**	V-type H+-transporting ATPase subunit B
**VHAG**	V-type H+-transporting ATPase subunit G
**VPACA**	V-type proton ATPase catalytic subunit A
